# Specific arrangements of species dominance can be more influential than evenness in maintaining ecosystem process and function

**DOI:** 10.1038/srep39325

**Published:** 2016-12-20

**Authors:** Daniel Wohlgemuth, Martin Solan, Jasmin A. Godbold

**Affiliations:** 1 Ocean and Earth Science, National Oceanography Centre Southampton, University of Southampton, Waterfront Campus, European Way, Southampton SO14 3ZH, UK; 2 Biological Sciences, University of Southampton, Highfield, Southampton, SO17 1BJ, UK

## Abstract

The ecological consequences of species loss are widely studied, but represent an end point of environmental forcing that is not always realised. Changes in species evenness and the rank order of dominant species are more widespread responses to directional forcing. However, despite the repercussions for ecosystem functioning such changes have received little attention. Here, we experimentally assess how the rearrangement of species dominance structure within specific levels of evenness, rather than changes in species richness and composition, affect invertebrate particle reworking and burrow ventilation behaviour - important moderators of microbial-mediated remineralisation processes in benthic environments - and associated levels of sediment nutrient release. We find that the most dominant species exert a disproportionate influence on functioning at low levels of evenness, but that changes in biomass distribution and a change in emphasis in species-environmental interactions become more important in governing system functionality as evenness increases. Our study highlights the need to consider the functional significance of alterations to community attributes, rather than to solely focus on the attainment of particular levels of diversity when safeguarding biodiversity and ecosystems that provide essential services to society.

Alterations to biodiversity influence the functioning of ecosystems and, by extension, the services that benefit human society, as evidenced by a plethora of experiments that have altered the number of genes, species or functional groups within a community and observed associated changes in ecosystem functioning and services^1^. However, the effects of natural or human-induced factors on biological communities are not solely limited to the adjustment of species richness, they also affect other important aspects of biodiversity, in particular species evenness^2^,^3^,^4^, the identity and rank order of dominant species^5^,^6^, and the spatial arrangement of individuals within a community^7^,^8^. Changes in such community attributes tend to depend on biotic and/or abiotic context across scales, from local habitat conditions^7^ to climatic forcing^2^, occur over extended timescales and are often a prelude to local species extinction^9^. Moreover, they modify the relative distribution of functional traits, the nature of species-environment relations and the strength of species interactions that are important for mediating ecosystem processes and functioning^7^,^10^,^11^.

Whilst theory predicts that increases in evenness will enhance synergistic interspecific interactions that intensify species contributions to ecosystem functioning^12^, empirical studies that have examined the effects of changes in evenness on ecosystem properties report mixed results (positive^13^,^14^; negative^15^,^16^; neutral^17^). Recent work, however, has shown that the functional outcome of a change in evenness can be attributed to substitutions in species composition^18^ and rearrangements in the order of species dominance^19^,^20^ rather than changes in evenness *per se*. Hence, when a community is dominated by a species that disproportionately contributes to functioning, a shift towards a more even community is more likely to promote species that are functionally inferior and lead to a decline in function^21^. Conversely, when a community is dominated by a species that contributes least to functioning, better performing species will increase in relative abundance as communities become more even and elevate the level of functioning^22^. Furthermore, such changes in dominance hierarchy may alter functionally important aspects of species interactions^14^. These can be antagonistic or synergistic depending on the identity and relative abundances of the interacting species^23^ and can lead to negative or positive consequences for ecosystem functioning^12^,^15^,^24^. Thus, it is the interplay between species dominance and the relative distribution of traits within a community that can be important in moderating ecosystem properties^25^,^26^, because multiple permutations of dominance structure are possible, and are likely to affect the strength and direction of species interactions, within each level of evenness.

Here, we determine whether alterations in the identity and rank order of dominant species across contrasting levels of community evenness affect benthic community contributions to ecosystem process (particle reworking and bioirrigation = bioturbation) and functioning (nutrient cycling). Our *a priori* expectation was that ecosystem properties would be higher and less variable in communities in which species are more evenly distributed, because the functional expression of species traits is more likely to balance and the probability of positive synergistic interactions would increase^27^. We also speculated that differences in dominance order would explain deviations from these predictions because the identity of the most dominant species will exert a disproportionate influence on net trait contributions to functioning. If our expectations are met, it raises the possibility that the use of simple diversity metrics to represent complex communities may form an insufficient vehicle for determining the functional integrity of an ecosystem^28^,^29^,^30^.

## Results

### Effects of evenness on ecosystem process and functioning

**Figure 1:**
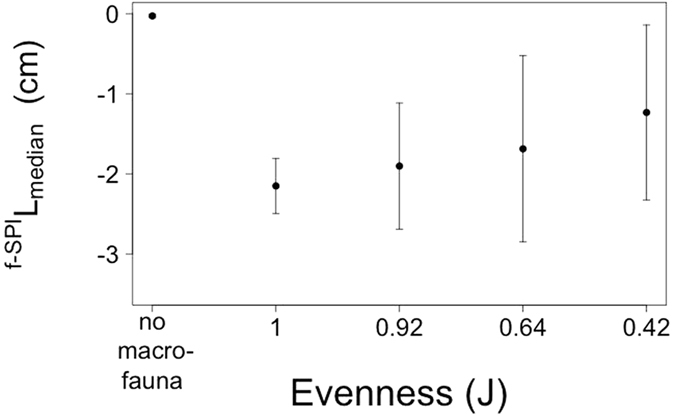
The effects of community evenness (J) on the mean depth of sediment particle reworking (^f-SPI^L_mean_, cm, mean ± s.d., n = 5) calculated from the vertical distribution of luminophore tracers (Supplementary Figure S2). Negative values indicate depth below the sediment-water interface. Observations in the absence of macrofauna are shown for comparison, but were not included in the statistical analyses.

We found no evidence that evenness, when treated as a continuous explanatory variable, affected the mean mixed depth of particle reworking (^f-SPI^L_mean_: F = 2.23, d.f. = 1, p = 0.140; Supplementary Model S1), maximum mixed depth of particle reworking (^f-SPI^L_max_: F = 0.04, d.f. = 1, p = 0.843; Supplementary Model S2), surface boundary roughness (SBR: F = 0.003, d.f. = 1, p = 0.956; Supplementary Model S3) or bioirrigation activity (Δ[Br^−^]: F = 0.17, d.f. = 1, p = 0.682; Supplementary Model S4). However, there was an effect of evenness on the median mixed depth of particle reworking (^f-SPI^L_median_: L-ratio = 4.37, d.f. = 1, p = 0.037; Supplementary Model S5, Fig. 1), ranging from (mean ± s.d.) 1.23 ± 1.09 cm for J^0.42^ to 2.15 ± 0.34 cm for J^1.00^.

**Figure 2:**
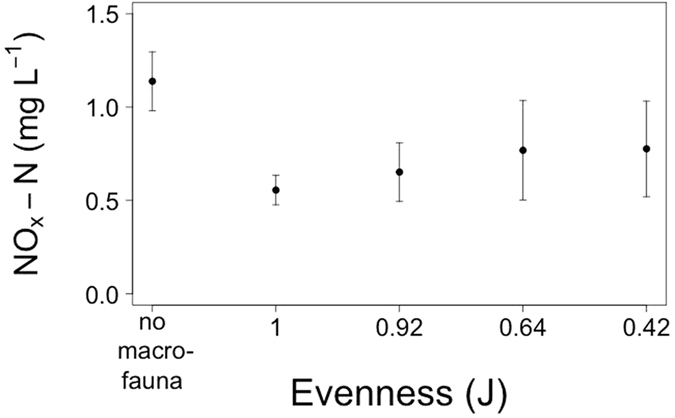
The effects of community evenness (J) on NO_X_-N concentrations (mg L^−1^, mean ± s.d., n = 5). Nutrient concentrations observed in the absence of macrofauna are shown for comparison, but were not included in the statistical analyses.

For nutrient concentrations, our analyses reveal that changes in species evenness did not influence [NH_4_-N] (F = 0.17, d.f. = 1, p = 0.681, Supplementary Model S6) or [PO_4_-P] (F = 2.90, d.f. = 1, p = 0.093, Supplementary Model S7), but [NO_x_-N] did decrease (mean ± s.d., from 0.78 ± 0.26 mg L^−1^ for J^0.42^ to 0.56 ± 0.08 mg L^−1^ for J^1.00^) with increased evenness (L-ratio = 8.25, d.f. = 1, p = 0.004, Supplementary Model S8, Fig. 2). Interestingly, our data also suggests that a shift towards greater evenness (J → 1) reduces variability (standard deviation) in the response variables (i.e. there is a reduction in standard deviation for: ^f-SPI^L_mean_, J^0.42^ = 0.88 → J^1.00^ = 0.60; ^f-SPI^L_median_, J^0.42^ = 1.09 → J^1.00^ = 0.34; ^f-SPI^L_max_, J^0.42^ = 0.58 → J^1.00^ = 0.29; SBR, J^0.42^ = 0.36 → J^1.00^ = 0.36; [NH_4_-N], J^0.42^ = 1.02 → J^1.00^ = 0.58; [NO_X_-N], J^0.42^ = 0.26 → J^1.00^ = 0.08). A reanalysis of our data using evenness as a nominal explanatory variable confirm these results (Supplementary Model S9–S16).

### Effects of changes in the arrangement of dominance structure on ecosystem process and functioning

**Figure 3:**
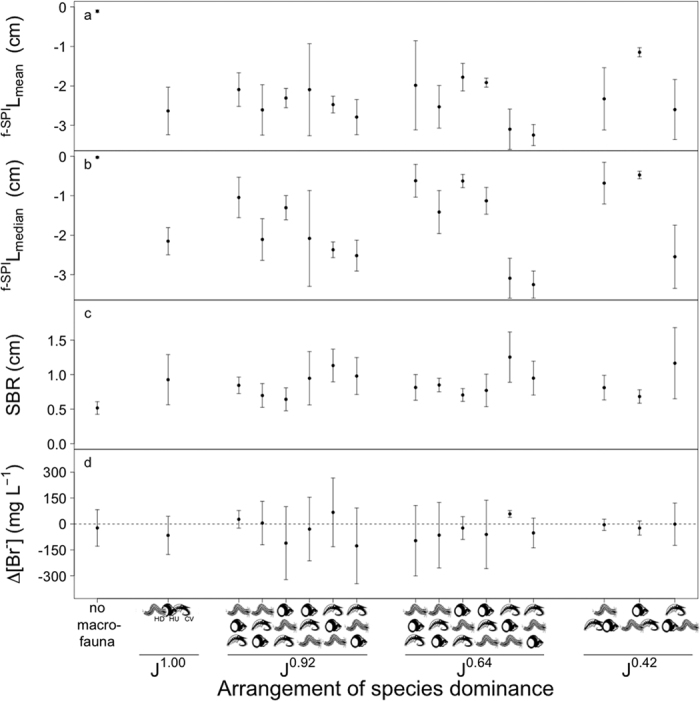
The effects of changes in the arrangement of species dominance within different evenness levels (J^1.00^, J^0.92^, J^0.64^, J^0.42^; Figure S1) on (**a**) the mean depth of sediment particle reworking (^f-SPI^L_mean_, cm, mean ± s.d., n = 5) and (**b**) the median depth of sediment particle reworking (^f-SPI^L_median_, cm, mean ± s.d., n = 5) calculated from the vertical distribution of luminophore tracers (Supplementary Figure S2), (**c**) the surface boundary roughness (SBR, cm, mean ± s.d., n = 5) and (**d**) bioirrigation activity (Δ[Br^−^] mg L^−1^, mean ± s.d., n = 5). For mean and median depth of sediment particle reworking and bioirrigation activity negative values indicate increased activity. Observations in the absence of macrofauna are shown for comparison, but were not included in the statistical analyses. The sequence of species dominance (vertically, from most to least; horizontally, equal dominance) is indicated on the x-axis. Species are represented graphically, and are indicated at J^1.00^ by abbreviation: HD = *Hediste diversicolor*, HU = *Hydrobia ulvae*, CV = *Corophium volutator*.

We found strong effects of changes in the arrangement of dominance structure on ecosystem process (Supplementary Model S17–S21) that were driven by the activities of *Corophium volutator*, followed by those of *Hediste diversicolor* and those of *Hydrobia ulvae*. The mean and median mixed depth of particle reworking (^f-SPI^L_mean_: L-ratio = 78.76, d.f. = 15, p = <0.0001, Supplementary Model S17; ^f-SPI^L_median_: F = 4.17, d.f. = 15, p = <0.0001, Supplementary Model S18) differed between alternative dominance structures, with the largest differences occurring at lower evenness levels (J^0.64^ and J^0.42^; Fig. 3). Treatments dominated by *Corophium volutator* (CV) and *Hediste diversicolor* (HD) tended to result in a greater degree of particle mixing (mean ± s.d.) relative to treatments where *Hydrobia ulvae* (HU) was dominant (^f-SPI^L_mean_: CV = 2.98 ± 0.58 cm, HD = 2.28 ± 0.82 cm, HU = 1.61 ± 0.40 cm; ^f-SPI^L_median_: CV = 2.96 ± 0.62 cm, HD = 0.91 ± 0.59 cm, HU = 0.74 ± 0.36 cm; Fig. 3). In addition, ^f-SPI^L_median_ was affected by the rank order of the subdominant species in treatments dominated by *Hediste diversicolor* for J^0.64^ and by *Hediste diversicolor* and *Hydrobia ulvae* for J^0.92^ (coefficient table, Supplementary Model S18), increasing when *Corophium volutator* was the second most dominant species in terms of biomass (Fig. 3). Surficial sediment reworking activities (SBR: L-ratio = 36.98, d.f. = 15, p = 0.001; Supplementary Model S19) were affected by alterations to dominance structure (Fig. 3), but the maximum depth of mixing was unaffected (^f-SPI^L_max_: F = 1.29, d.f. = 15, p = 0.237; Supplementary Model S20). Bioirrigation activity (Δ[Br^−^]) was also affected by alternative arrangements of dominance structure, (L-ratio = 26.06, d.f. = 15, p = 0.037; Supplementary Model S21), but the sequence of species-specific effects was not as pronounced as the patterns observed for particle reworking (Fig. 3).

**Figure 4:**
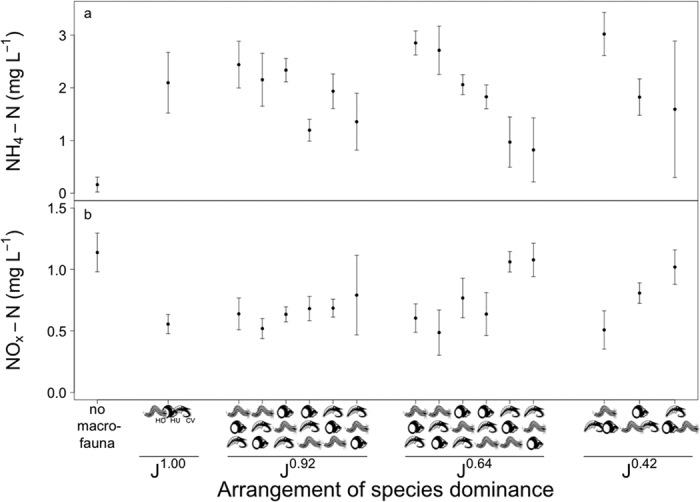
The effects of changes in the arrangement of species dominance within different evenness levels (J^1.00^, J^0.92^, J^0.64^, J^0.42^; Figure S1) on water column nutrient concentration (mg L^−1^, mean ± s.d., n = 5) for (**a**) [NH_4_-N] and (**b**) [NO_x_-N]. Nutrient concentrations observed in the absence of macrofauna are shown for comparison, but were not included in the statistical analyses. The sequence of species dominance (vertically, from most to least; horizontally, equal dominance) is indicated on the x-axis. Species are represented graphically, and are indicated at J^1.00^ by abbreviation: HD = *Hediste diversicolor*, HU = *Hydrobia ulvae*, CV = *Corophium volutator*.

Alterations in the arrangement of species dominance also led to changes in nitrogen cycling ([NH_4_-N]: L-ratio = 79.21, d.f. = 15, p < 0.0001, Supplementary Model S22; [NO_x_-N]: F = 8.53, d.f. = 15, p < 0.0001, Supplementary Model S23; Fig. 4), but not for [PO_4_-P] (F = 0.57, d.f. = 15, p = 0.889, Supplementary Model S24). At the lowest levels of evenness, [NH_4_-N] (mean ± s.d.) was low when *Corophium volutator* was dominant (1.59 ± 1.29 mg L^−1^ for J^0.42^), intermediate when treatments were dominated by *Hydrobia ulvae* (1.82 ± 0.35 mg L^−1^ for J^0.42^) and highest when *Hediste diversicolor* was dominant (3.02 ± 0.41 mg L^−1^ for J^0.42^). The [NO_X_-N] were reciprocal to those of [NH_4_-N], suggesting a predominance of denitrification, with lowest concentrations (mean ± s.d.) for treatments dominated by *Hediste diversicolor* (0.51 ± 0.16 mg L^−1^ for J^0.42^) followed by *Hydrobia ulvae* (0.81 ± 0.8 mg L^−1^ for J^0.42^) and *Corophium volutator* (1.02 ± 0.14 mg L^−1^ for J^0.42^). This pattern was largely maintained at intermediate levels of dominance (J^0.64^), but was less prominent at higher levels of dominance (J^0.92^ and J^1.00^, Fig. 4). At high levels of evenness (J^0.92^) [NH_4_-N] was lower when *Corophium volutator* was the second most dominant species after *Hydrobia ulvae* (coefficient table Supplementary Model S22, Fig. 4).

## Discussion

We have demonstrated that, irrespective of evenness level, rearrangements in the rank order of species dominance can lead to distinct changes in ecosystem properties that, in turn, depend on the functional identity of the most dominant species. This implies that differences in species dominance might best explain apparent inconsistent community responses to directional changes in evenness^13^,^14^,^15^,^16^,^17^,^31^, although the observed levels of nutrient concentrations in our experiments were not always coherent with bioturbation^32^. Such discrepancies are maximised at low levels of evenness where the most dominant species exert a disproportionate influence on functioning, but increasingly reflect changes in biomass distribution and the role of less abundant species as evenness increases. These effects can be augmented, if synergistic interspecific interactions are mediated by alterations in species biomass^10^, or if response variables may be functionally divergent in their sensitivities to species traits and interactions^33^. Furthermore, species contributions to ecosystem properties can reflect a range of simultaneously operating mechanisms that are not necessarily proportional to species biomass^34^. Changes in species behaviour^35^, density^36^, excretion^37^ and/or the architecture of biogenic features (mounds, pits, tubes and burrow galleries^38^), for example, can disproportionately influence microbial community structure and associated biochemical transformations^39^. Indeed, for nitrogen, divergence in the relative contributions of particle reworking, bioirrigation and nutrient generation observed here do suggest that alterations in the nature of species interactions and/or the expression of traits accompanied changes in evenness. Interestingly, this was not the case for phosphate, where changes in the arrangement of species dominance had little influence. Complex chemical retention systems can decouple species traits from aspects of nutrient release and may, under certain circumstances, overwhelm biotic control^40^,^41^. Nevertheless, our findings generally indicate that the functional outcome of a change in evenness is dependent not only on the arrangement of dominance structure, the realised density of individual species, and the rank order of less abundant species, but also on the propensity of species to adjust their functional role under novel biotic and/or abiotic circumstances^7^,^35^,^42^.

A second prominent outcome from our findings is that we found little evidence to support the view that changing levels of evenness facilitate synergistic interspecific interactions^27^,^43^. Instead, an increase in evenness led to a reduction in variance and a convergence in ecosystem performance that reflected interspecific alterations in biomass. Previous experimental work emphasised that ecosystem properties tend to be maximised by the traits of individual species^5^,^44^,^45^,^46^ and that interspecific synergistic interactions are unlikely, at least initially, as complex interactions between combinations of species and resources underlie mechanisms of complementarity and take time to develop^42^,^47^. However, the relative importance and nature (synergistic versus antagonistic) of interspecific interactions depends on the identity of the interacting species^23^, the identity of the response variable, and is further complicated by alterations in context, including resource availability^48^, habitat configuration^7^ and changing environmental conditions^42^,^49^,^50^. It follows therefore, that the mechanistic basis of species interactions are unlikely to be documented in short-term experiments, but will be more prominent in naturally assembled systems where there is a multi-generational history of species interaction^47^.

It is important to consider our findings within the context of natural ecosystems. Skewed species-abundance distributions, where only a few species dominate amongst many rare species, are a universal feature of biological communities^36^,^51^ and can constrain any effect of biodiversity on ecosystem functioning to a subset of dominant species. Whilst a shift in the identity of the most dominant species can lead to considerable changes in net community contribution to ecosystem properties^25^,^36^, the importance of less abundant or rare species cannot be disregarded as has been highlighted here and elsewhere^52^,^53^. However, communities in natural systems are not isolated and interact with other communities within the regional species pool, leading to complex meta-community dynamics^54^. When meta-communities are dominated by a single species, substitution of the most dominant species is likely to have a strong local impact on ecosystem functioning. In contrast, when meta-community populations are dominated by a range of different species, regional evenness is correspondingly elevated^12^, leading to reduced variability in ecosystem properties that, in turn, acts to stabilise functioning against shifts in dominance arrangement^55^. It may be anticipated, therefore, that evenness will be especially important at larger scales when environmental fluctuations^5^,^56^ and the multifunctionality of ecosystems are considered^57^, a view that places emphasis on the reciprocal relationship between biodiversity and the environment^58^.

Overall, our findings are consistent with current consensus based on small scale experiments that both the identity and the diversity of organisms jointly control the functioning of ecosystems^1^ and that species identity effects and community composition are most important, but we acknowledge that species richness and community biomass may become more important at larger scales^59^. Nevertheless, our study highlights the need to consider the functional significance of changes in the properties of biodiversity, rather than solely focus on the attainment or maintenance of biodiversity *per se*. In particular, more emphasis is required on the distribution of functional traits across different spatial scales, temporal variation in species contributions to ecosystem properties and variability in trait expression in changing environments. Such information will be essential if we are to guide efforts to protect species and ecosystem services or generate ecosystem models that accurately predict the ecological consequences of environmental change.

## Methods

Surficial sediment (less than 3 cm depth) and individuals of the gastropod *Hydrobia ulvae* and mud shrimp *Corophium volutator* were collected by sieving from the Hamble Estuary, Southampton (50°53′20.2″N 1°17′35.3″W), whilst individuals of the polychaete *Hediste diversicolor* were collected by hand from Langstone Harbour, Portsmouth (50°50′46.5″N 1°00′05.3″W) during April 2014. Sediment was sieved (500 μm mesh) in a seawater bath to remove macrofauna, allowed to settle for 48 h (to retain the fine fraction, <63 μm) and homogenised.

We assembled replicate (n = 5) macrofaunal communities across four evenness levels (Pielou’s evenness index, J;^60^) that span the spectrum of dominance curves theoretically possible in natural communities (J = 0.47–0.78^61^,^62^) by altering the distribution of biomass (constrained to 2.0 g aquarium^−1^). We based our treatments on biomass rather than species abundance as biomass is regarded to better integrate species functional traits that are important for bioturbation^63^. Specifically, communities were established in which species either had identical biomass (J^1.00^), the biomass of each species decreased sequentially in equal proportions (J^0.92^), or a single species dominates and the remaining biomass levels decrease either linearly (J^0.64^) or are held constant (J^0.42^) (Supplementary Figure S1). To allow the generality of any evenness effects to be evaluated, whilst enabling the identification of any effects caused by differences in the relative distribution of individual species, all possible permutations of species dominance order (J^1.00^, 1 permutation; J^0.92^, 6 permutations; J^0.64^, 6 permutations; J^0.42^, 3 permutations) were assembled (Supplementary Table S2). As nutrient cycling is primarily a microbial process, aquaria containing no macrofauna (n = 5) were included to distinguish the extent of macrofaunal mediation. As our focus was to determine the effect of altered levels of evenness and dominance, rather than presence versus absence effects, these aquaria were not included in the statistical analysis. The experimental design required a total of 85 aquaria (16 permutations of species dominance + aquaria containing no macrofauna x 5 replicates).

We used transparent square acrylic aquaria (internal dimensions, LWH; 12 × 12 × 35 cm), filled to 10 cm with mud and overlain by 20 cm of seawater (UV sterilised, 10 μm filtered, salinity 33). Seawater was replaced after 24 h to remove excess nutrients associated with assembly. Aquaria were randomly positioned in a recirculating seawater bath at 10 ± 1 °C under a 12:12 h light regime (Aqualine T5 Reef White 10 K fluorescent light tubes, Aqua Medic) and continually aerated for 12 days.

Faunal mediated sediment particle reworking was estimated non-invasively using a sediment profile imaging camera (Canon 400D, set to 30 s exposure, aperture f4.5 and ISO 400; 3888 × 2592 pixels, effective resolution at aquarium side = 57 × 57 μm per pixel), optically modified to allow preferential imaging of fluorescently labelled particulate tracers (luminophores, red colour, size class less than 125 μm; Brianclegg Ltd., UK) under UV light (f-SPI^64^) that were introduced on the first day of the experiment (25 g aquarium^−1^). Vertical luminophore particle re-distribution was determined from stitched composite images (RGB colour, JPEG compression, GNU Image Manipulation Program, Version 2.8.4, http://www.gimp.org/, Kimball, S., Mattis, P., GIMP (1995), Date of access 01/07/2014) of all four sides of each aquarium obtained in a UV illuminated imaging box^65^ using a custom-made semi-automated macro that runs within ImageJ (Version 1.47), a java-based public domain program developed at the US National Institutes of Health (http://rsb.info.nih.gov/ij/index.html, Rasband, W., ImageJ., (1997), Date of access 01/07/2014). From these data, the mean (^f-SPI^L_mean_, time dependent indication of mixing), median (^f-SPI^L_median_, typical short-term depth of mixing) and maximum (^f-SPI^L_max_, maximum extent of mixing over the long-term) mixed depth of particle redistribution were calculated. In addition, the vertical deviation of the sediment-water interface (upper – lower limit; surface boundary roughness, SBR) provided an indication of surficial activity^38^.

Following the addition of 2.74 g of the inert tracer sodium bromide (NaBr, dissolved in 10 ml seawater, = ~9 mM aquaria^−1^), bioirrigation was estimated from absolute changes in the concentration of bromide (∆[Br^−^], mg L^−1^; negative values indicate increased bioirrigation activity) over a 4 h period^66^ on day 12, determined from pre-filtered (Fisherbrand, QL100, Ø 70 mm) water samples (5 ml, taken centrally and ~5 cm above the sediment-water interface) using a flow injection auto-analyser (FIAstar 5000 series, Foss-Tecator).

Nutrient concentrations (ammonium, NH_4_-N; nitrate + nitrite, NO_x_-N; and phosphate, PO_4_-P) were determined from pre-filtered (Fisherbrand, nylon 0.45 μm, Ø 25 mm) water samples (10 ml, taken centrally and ~2 cm below the air-water interface) taken on day 12 by flow injection auto-analysis (FIA Star 5010 series) using an artificial seawater carrier solution.

We developed two separate statistical models for each of the dependent variables (ecosystem processes: ^f-SPI^L_mean_, ^f-SPI^L_median_, ^f-SPI^L_max_, SBR, Δ[Br^−^]; ecosystem functioning: [NH_4_-N], [NO_x_-N], [PO_4_-P]), to establish the independent effect of evenness *per se* and the effect of alterations in the arrangement of species dominance. We treated evenness as a continuous explanatory variable (to establish generic effects of evenness *per se*) and, in an alternative analysis, as a single nominal explanatory variable (to establish specific effects of our 4 treatment levels: J^1.00^, J^0.92^, J^0.64^, J^0.42^). Species dominance was treated as a single nominal explanatory variable (16 levels, Supplementary Table S2).

The initial linear regression models were assessed for normality (Q-Q-plot), heterogeneity of variance (plotted residual vs. fitted values) and influential data points (cook’s distance)^67^. Where data exploration indicated heterogeneity of variance due to differences in the number of evenness permutations per treatment level, we allowed the residual spread to vary with evenness using generalized least squares (GLS) estimation. This procedure uses appropriate variance functions (here *varIdent* for nominal and *varPower* or *varExp* for continuous explanatory variables) to model the variance structure^67^. The optimal variance covariate structure was determined by comparing the initial regression model without variance structure to the equivalent GLS model incorporating specific variance structures using AIC and visualisation of model residuals following restricted maximum likelihood (REML) estimation. The optimal fixed structure was determined by applying backward selection using the likelihood ratio test obtained by maximum likelihood (ML) estimation^67^. The coefficient tables are presented (Supplementary information, statistical model summary, Model S1–S24) without correction for the alpha-error, as Bonferroni correction increases the beta error and tends to obscure multiple significant results if p-values are moderate and the statistical power is low^68^. Data in the figures are presented as mean ± standard deviation for all response variables, as we wanted to visualise the spread and variability of the data. All statistical analysis were performed using the ‘R’ statistical and programming environment^69^ and the “nlme” package^70^. Data used in our statistical analyses are provided in Supplementary Table S1.

## Additional Information

**How to cite this article**: Wohlgemuth, D. *et al*. Specific arrangements of species dominance can be more influential than evenness in maintaining ecosystem process and function. *Sci. Rep.* **6**, 39325; doi: 10.1038/srep39325 (2016).

**Publisher's note:** Springer Nature remains neutral with regard to jurisdictional claims in published maps and institutional affiliations.

## Supplementary Material

Supplementary Information
