# Supplementary material for: Specific arrangements of species dominance can be more influential than evenness in maintaining ecosystem process and function
Source: Sci Rep. 2016 Dec 20;6:39325. doi: 10.1038/srep39325 (PMC5171799; doi:10.1038/srep39325)
Supplement: Supplementary Information [file srep39325-s1.pdf]

Supplementary information

Specific arrangements of species dominance can be more influential than evenness in maintaining ecosystem process and function

Daniel Wohlgemuth[1], Martin Solan[1], Jasmin A. Godbold[1,2]

[1]Ocean and Earth Science, National Oceanography Centre Southampton, University of Southampton, Waterfront Campus, European Way, Southampton SO14 3ZH, UK,

[2] Biological Sciences, University of Southampton, Highfield, Southampton, SO17 1BJ, UK.

Correspondence and requests for materials should be addressed to D.W. (email: d.wohlgemuth@soton.ac.uk)

Statistical model summary

Summary of the statistical models (Model S1 to S24). For Model S1 to S8 evenness is treated as a continuous independent variable and for Model S9 to S16 as a categorical independent variable. For each model, we list the initial linear regression model and the minimal adequate model. Where it was necessary to account for a violation of homogeneity of variance, we used a linear regression with GLS estimation and we provide a summary of the coefficient table. The coefficients indicate the relative performance of each treatment level relative to the relevelled baseline (as indicated). Coefficients ± SE, t-values and respective significance values are presented. Levels of significance for $p<0.05$, $p<0.01$ and $p<0.001$ are highlighted in grey shading (darker shading with increasing significance). Abbreviations: HD, *Hediste diversicolor*; HU, *Hydrobia ulvae*; CV, *Corophium volutator*.

Statistical models for the effects of evenness

(i) Evenness treated as a continuous variable

Model S1: Mean mixed depth of particle reworking ($^{f\text{-}SPI}L_{mean}$, cm)

Initial linear regression model:

Lm($^{f\text{-}SPI}L_{mean}$~ J)

No minimal adequate model, intercept only (J, F = 2.23, d.f. = 1, p = 0.140)

Model S2: Maximum mixed depth of particle reworking ($^{f\text{-}SPI}L_{max}$, cm)

Initial linear regression model:

Lm($^{f\text{-}SPI}L_{max}$~ J)

No minimal adequate model, intercept only (J, F = 0.04, d.f. = 1, p = 0.84)

Model S3: Surface boundary roughness (SBR, cm)

Initial linear regression model:

Lm(SBR~ J)

No minimal adequate model, intercept only (J, F = 0.003, d.f. = 1, p = 0.956)

Model S4: Bioirrigation ($\Delta[Br^-]$, mg L^{-1})

Initial linear regression model:

Lm($\Delta[Br^-]$ ~ J)

No minimal adequate model, intercept only (J, F = 0.17, d.f. = 1, p = 0.68)

Model S5: Median mixed depth of particle reworking ($^{f\text{-}SPI}L_{median}$, cm)

Initial linear regression model:

Lm($^{f\text{-}SPI}L_{median}$~ J)

Minimal adequate model:

gls($^{f\text{-}SPI}L_{median}$~ J, weights = varExp (form = ~ J), method = 'ML')

(J, L-ratio = 4.37, d.f. = 1, p = 0.037)

Coefficient table (method = 'REML'):

	**Coefficient**	**± SE**	**t-value**	**p**
**Intercept**	0.848	0.467	1.817	0.073
**Slope (J)**	1.195	0.567	2.105	0.039

Model S6: NH_4-N concentration ([NH_4-N], mg L^{-1})

Initial linear regression model:

Lm([NH_4-N] ~ J)

No minimal adequate model, intercept only (J, F = 0.17, d.f. = 1, p = 0.68)

Model S7: PO_4-P concentration ([PO_4-P], mg L^{-1})

Initial linear regression model:

Lm([PO_4-P] ~ J)

No minimal adequate model, intercept only (J, F = 2.90, d.f. = 1, p = 0.093)

Model S8: NO_x-N concentration ([NO_x-N], mg L^{-1})

Initial linear regression model:

Lm([NO_x-N] ~ J)

Minimal adequate model:

gls([NO_x-N] ~ J, weights = varExp(form = ~ J), method = 'ML')

(J, L-ratio = 8.25, d.f. = 1, p = 0.004)

Coefficient table (method = 'REML'):

	Coefficient	± SE	t-value	p
**Intercept**	0.995	0.111	8.982	<0.0001
**Slope (J)**	-0.384	0.131	-2.919	0.005

(ii) Evenness treated as nominal variable

Model S9: Mean mixed depth of particle reworking ($^{f\text{-}SPI}L_{mean}$, cm)

Initial linear regression model:

Lm($^{f\text{-}SPI}L_{mean}$~ J)

No minimal adequate model, intercept only (J, F = 1.36, d.f. = 3, p = 0.262)

Model S10: Max mixed depth of particle reworking ($^{f\text{-}SPI}L_{max}$, cm)

Initial linear regression model:

Lm($^{f\text{-}SPI}L_{max}$~ J)

No minimal adequate model, intercept only (J, F = 0.064, d.f. = 3, p = 0.979)

Model S11: Surface boundary roughness (SBR, cm)

Initial linear regression model:

Lm(SBR~ J)

No minimal adequate model, intercept only (J, F = 0.05, d.f. = 3, p = 0.985)

Model S12: Bioirrigation ($\Delta[Br^-]$, mg L^{-1})

Initial linear regression model:

Lm($\Delta[Br^-]$ ~ J)

No minimal adequate model, intercept only (J, L-ratio = 1.84, d.f. = 3, p = 0.864)

Model S13: Median mixed depth of particle reworking (${}^{f\text{-}SPI}L_{median}$, cm)

Initial linear regression model:

Lm(${}^{f\text{-}SPI}L_{median}$~ J)

Minimal adequate model:

gls(${}^{f\text{-}SPI}L_{median}$~ J, weights = varIdent(form = ~1|J), method = 'ML')

(J, L-ratio = 8.49, d.f. = 3, p = 0.037)

Coefficient table (method = 'REML'): Intercept ± SE (For baseline J = 1): 2.151 ± 0.154, t = 13.988, p = <0.0001. Coefficients ± SE and t-values are presented. Significance values are in bold.

	$J^{1.00}$	$J^{0.92}$	$J^{0.64}$	$J^{0.42}$
$J^{0.92}$	-0.248 ± 0.211<br>-1.177<br>**0.243**			
$J^{0.64}$	-0.463 ± 0.262<br>-1.767<br>**0.081**	-0.215 ± 0.256<br>-0.839<br>**0.404**		
$J^{0.42}$	-0.917 ± 0.321<br>-2.855<br>**0.006**	-0.669 ± 0.317<br>-2.114<br>**0.038**	-0.454 ± 0.353<br>-1.286<br>**0.202**	

Model S14: NH_4-N concentration ([NH_4-N], mg L^{-1})

Initial linear regression model:

Lm([NH_4-N] ~ J)

No minimal adequate model, intercept only (J, F = 0.49, d.f. = 3, p = 0.691)

Model S15: PO_4 concentration ([PO_4-P], mg L^{-1})

Initial linear regression model:

Lm([PO_4-P] ~ J)

No minimal adequate model, intercept only (J, F = 1.2, d.f. = 3, p = 0.317)

Model S16: NO_x-N concentration ([NO_x-N], mg L^{-1})

Initial linear regression model:

Lm([NO_x-N] ~ J)

Minimal adequate model:

gls([NO_x-N] ~ J, weights = varIdent(form = ~1|J), method = 'ML')

(J, L-ratio = 12.92, d.f. = 3, p = 0.005)

Coefficient table (method 'REML'): Intercept ± SE (For baseline J = 1): 0.556 ± 0.035, t = 15.761, p = <0.0001. Coefficients ± SE and t-values are presented. Significance values are in bold.

	**$J^{1.00}$**	**$J^{0.92}$**	**$J^{0.64}$**	**$J^{0.42}$**
**$J^{0.92}$**	0.096 ± 0.046<br>2.073<br>**0.042**			
**$J^{0.64}$**	0.213 ± 0.061<br>3.469<br>**0.001**	0.117 ± 0.059<br>1.987<br>**0.051**		
**$J^{0.42}$**	0.221 ± 0.077<br>2.867<br>**0.006**	0.124 ± 0.075<br>1.663<br>**0.101**	0.008 ± 0.085<br>0.093<br>**0.927**	

Models for the effects of specific arrangements of species dominance (SpD)

Model S17: Mean mixed depth of particle reworking ($^{f\text{-}SPI}L_{mean}$, cm)

Initial linear regression model:

Lm($^{f\text{-}SPI}L_{mean}$~ SpD)

Minimal adequate model:

gls($^{f\text{-}SPI}L_{mean}$~ SpD, weights = varIdent (form = ~ 1|SpD), method = 'ML')

(SpD, L-ratio = 78.76, d.f. = 15, p = <0.0001)

Coefficient table model S17 (method = 'REML'): Intercept ± SE (For baseline J = 1): 2.633 ± 0.269, t = 9.807, p = <0.0001. Coefficients ± SE and t-values are presented. Significance values are in bold. CV = *Corophium volutator*, HD = *Hediste diversicolor*, HU = *Hydrobia ulvae*

	$J^{1.00}$	$J^{0.64}$ CV>HD>HU	$J^{0.92}$ CV>HD>HU	$J^{0.64}$ CV>HU>HD	$J^{0.92}$ CV>HU>HD	$J^{0.42}$ CV>HU=HD	$J^{0.64}$ HD>HU>CV	$J^{0.92}$ HD>HU>CV	$J^{0.64}$ HD>CV>HU	$J^{0.92}$ HD>CV>HU	$J^{0.42}$ HD>CV=HU	$J^{0.64}$ HU>CV>HD	$J^{0.92}$ HU>CV>HD	$J^{0.64}$ HU>HD>CV	$J^{0.92}$ HU>HD>CV
$J^{0.64}$ CV>HD>HU	0.611 ± 0.294<br>2.078<br>**0.042**														
$J^{0.92}$ CV>HD>HU	0.156 ± 0.334<br>0.466<br>**0.643**	-0.455 ± 0.232<br>-1.966<br>**0.054**													
$J^{0.64}$ CV>HU>HD	0.463 ± 0.353<br>1.314<br>**0.194**	-0.147 ± 0.258<br>-0.571<br>**0.570**	0.308 ± 0.303<br>1.017<br>**0.313**												
$J^{0.92}$ CV>HU>HD	-0.161 ± 0.285<br>-0.564<br>**0.575**	-0.772 ± 0.152<br>-5.061<br>**<0.0001**	-0.316 ± 0.220<br>-1.440<br>**0.155**	-0.624 ± 0.248<br>-2.521<br>**0.014**											
$J^{0.42}$ CV>HU=HD	-0.034 ± 0.433<br>-0.077929<br>**0.9381**	-0.645 ± 0.360<br>-1.790069<br>**0.078**	-0.189 ± 0.393<br>-0.482<br>**0.632**	-0.497 ± 0.410<br>-1.214<br>**0.229**	0.127 ± 0.353<br>0.360<br>**0.720**										
$J^{0.64}$ HD>HU>CV	-0.649 ± 0.571<br>-1.136<br>**0.260**	-1.260 ± 0.518<br>-2.433<br>**0.018**	-0.804 ± 0.541<br>-1.486<br>**0.142**	-1.112 ± 0.553<br>-2.010<br>**0.049**	-0.488 ± 0.513<br>-0.952<br>**0.345**	-0.615 ± 0.608<br>-1.012<br>**0.315**									
$J^{0.92}$ HD>HU>CV	-0.541 ± 0.329<br>-1.643<br>**0.105**	-1.152 ± 0.225<br>-5.122<br>**<0.0001**	-0.696 ± 0.275<br>-2.533<br>**0.014**	-1.004 ± 0.298<br>-3.374<br>**0.001**	-0.380 ± 0.213<br>-1.787<br>**0.079**	-0.507 ± 0.389<br>-1.302<br>**0.198**	0.108 ± 0.539<br>0.200<br>**0.842**								
$J^{0.64}$ HD>CV>HU	-0.104 ± 0.360<br>-0.289<br>**0.773**	-0.715 ± 0.269<br>-2.663<br>**0.010**	-0.260 ± 0.312<br>-0.834<br>**0.407**	-0.568 ± 0.332<br>-1.711<br>**0.092**	0.056 ± 0.258<br>0.2181<br>**0.828**	-0.071 ± 0.416<br>-0.170<br>**0.866**	0.544 ± 0.558<br>0.975<br>**0.333**	0.436 ± 0.307<br>1.423<br>**0.160**							
$J^{0.92}$ HD>CV>HU	-0.026 ± 0.392<br>-0.067<br>**0.947**	-0.637 ± 0.309<br>-2.060<br>**0.044**	-0.182 ± 0.347<br>-0.524<br>**0.602**	-0.490 ± 0.366<br>-1.339<br>**0.185**	0.134 ± 0.301<br>0.447<br>**0.656**	0.007 ± 0.444<br>0.017<br>**0.987**	0.622 ± 0.579<br>1.075<br>**0.286**	0.514 ± 0.343<br>1.500<br>**0.139**	0.078 ± 0.373<br>0.209<br>**0.835**						
$J^{0.42}$ HD>CV=HU	-0.306 ± 0.443<br>-0.693<br>**0.491**	-0.917 ± 0.371<br>-2.469<br>**0.016**	-0.462 ± 0.404<br>-1.145<br>**0.257**	-0.770 ± 0.420<br>-1.835<br>**0.071**	-0.146 ± 0.364<br>-0.400<br>**0.690**	-0.273 ± 0.489<br>-0.558<br>**0.579**	0.342 ± 0.614<br>0.557<br>**0.580**	0.234 ± 0.400<br>0.586<br>**0.560**	-0.202 ± 0.426<br>-0.474<br>**0.637**	-0.280 ± 0.453<br>-0.619<br>**0.538**					
$J^{0.64}$ HU>CV>HD	-0.718 ± 0.273<br>-2.626<br>**0.011**	-1.329 ± 0.130<br>-10.222<br>**<0.0001**	-0.873 ± 0.205<br>-4.265<br>**0.0001**	-1.181 ± 0.234<br>-5.040<br>**<0.0001**	-0.557 ± 0.107<br>-5.181<br>**<0.0001**	-0.684 ± 0.344<br>-1.991<br>**0.051**	-0.069 ± 0.506<br>-0.136<br>**0.892**	-0.177 ± 0.197<br>-0.897<br>**0.373**	-0.613 ± 0.246<br>-2.495<br>**0.015**	-0.691 ± 0.290<br>-2.386<br>**0.020**	-0.411 ± 0.355<br>-1.157<br>**0.252**				
$J^{0.92}$ HU>CV>HD	-0.538 ± 0.586<br>-0.918<br>**0.362**	-1.149 ± 0.534<br>-2.150<br>**0.035**	-0.694 ± 0.557<br>-1.245<br>**0.218**	-1.001 ± 0.569<br>-1.760<br>**0.083**	-0.377 ± 0.529<br>-0.713<br>**0.479**	-0.504 ± 0.622<br>-0.811<br>**0.421**	0.111 ± 0.725<br>0.153<br>**0.879**	0.003 ± 0.555<br>0.005<br>**0.996**	-0.434 ± 0.574<br>-0.756<br>**0.453**	-0.512 ± 0.594<br>-0.862<br>**0.392**	-0.231 ± 0.629<br>-0.368<br>**0.714**	0.180 ± 0.523<br>0.343<br>**0.733**			
$J^{0.64}$ HU>HD>CV	-0.854 ± 0.310<br>-2.752<br>**0.008**	-1.465 ± 0.196<br>-7.468<br>**<0.0001**	-1.010 ± 0.252<br>-4.006<br>**0.0002**	-1.317 ± 0.277<br>-4.763<br>**<0.0001**	-0.693 ± 0.182<br>-3.809<br>**0.0003**	-0.820 ± 0.374<br>-2.195<br>**0.032**	-0.205 ± 0.527<br>-0.390<br>**0.698**	-0.313 ± 0.246<br>-1.274<br>**0.207**	-0.750 ± 0.286<br>-2.618<br>**0.011**	-0.828 ± 0.325<br>-2.548<br>**0.013**	-0.548 ± 0.385<br>-1.424<br>**0.159**	-0.136 ± 0.164<br>-0.833<br>**0.408**	-0.316 ± 0.544<br>-0.581<br>**0.563**		
$J^{0.92}$ HU>HD>CV	-0.327 ± 0.290<br>-1.127<br>**0.264**	-0.938 ± 0.162<br>-5.780<br>**<0.0001**	-0.483 ± 0.227<br>-2.129<br>**0.037**	-0.790 ± 0.254<br>-3.115<br>**0.003**	-0.166 ± 0.145<br>-1.147<br>**0.256**	-0.293 ± 0.357<br>-0.821<br>**0.415**	0.322 ± 0.516<br>0.624<br>**0.535**	0.214 ± 0.220<br>0.973<br>**0.334**	-0.223 ± 0.264<br>-0.842<br>**0.403**	-0.301 ± 0.306<br>-0.983<br>**0.329**	-0.020 ± 0.368<br>-0.055<br>**0.956**	0.391 ± 0.121<br>3.230<br>**0.002**	0.211 ± 0.532<br>0.397<br>**0.693**	0.527 ± 0.190<br>2.770<br>**0.007**	
$J^{0.42}$ HU>HD=CV	-1.486 ± 0.273<br>-5.438<br>**<0.0001**	-2.097 ± 0.130<br>-16.127<br>**<0.0001**	-1.642 ± 0.205<br>-8.017<br>**<0.0001**	-1.950 ± 0.234<br>-8.319<br>**<0.0001**	-1.326 ± 0.108<br>-12.323<br>**<0.0001**	-1.453± 0.344<br>-4.228<br>**0.0001**	-0.839 ± 0.506<br>-1.655<br>**0.103**	-0.946 ± 0.197<br>-4.796<br>**<0.0001**	-1.382 ± 0.246<br>-5.621<br>**<0.0001**	-1.460 ± 0.290<br>-5.038<br>**<0.0001**	-1.180 ± 0.355<br>-3.320<br>**0.002**	-0.769 ± 0.072<br>-10.633<br>**<0.0001**	-0.948 ± 0.523<br>-1.812<br>**0.075**	-0.632 ± 0.164<br>-3.863<br>**0.0003**	-1.160 ± 0.121<br>-9.579<br>**<0.0001**

$J^{0.42}$ HU>HD=CV

Model S18: Median mixed depth of particle reworking ($^{f\text{-}SPI}L_{median}$, cm)

Initial linear regression model:

Lm($^{f\text{-}SPI}L_{median}$~ SpD)

Minimal adequate model:

Lm($^{f\text{-}SPI}L_{median}$~ SpD)

(SpD, $F = 4.17$, d.f. = 15, $p = <0.0001$)

Coefficient table model S18 (method = 'REML'): 2.151 ± 0.233, t = 9.251, p = <0.0001. Coefficients ± SE and t-values are presented. Significance values are in bold. CV = *Corophium volutator*, HD = *Hediste diversicolor*, HU = *Hydrobia ulvae*

	$J^{1.00}$	$J^{0.64}$ CV>HD>HU	$J^{0.92}$ CV>HD>HU	$J^{0.64}$ CV>HU>HD	$J^{0.92}$ CV>HU>HD	$J^{0.42}$ CV>HU=HD	$J^{0.64}$ HD>HU>CV	$J^{0.92}$ HD>HU>CV	$J^{0.64}$ HD>CV>HU	$J^{0.92}$ HD>CV>HU	$J^{0.42}$ HD>CV=HU	$J^{0.64}$ HU>CV>HD	$J^{0.92}$ HU>CV>HD	$J^{0.64}$ HU>HD>CV	$J^{0.92}$ HU>HD>CV	$J^{0.42}$ HU>HD=CV
$J^{0.64}$ CV>HD>HU	1.099 ± 0.329<br>3.341<br>**0.001**															
$J^{0.92}$ CV>HD>HU	0.366 ± 0.329<br>1.112<br>**0.270**	-0.733 ± 0.329<br>-2.229<br>**0.029**														
$J^{0.64}$ CV>HU>HD	0.937 ± 0.329<br>2.850<br>**0.006**	-0.161 ± 0.329<br>-0.490<br>**0.625**	0.572 ± 0.329<br>1.739<br>**0.087**													
$J^{0.92}$ CV>HU>HD	0.216 ± 0.329<br>0.658<br>**0.513**	-0.882 ± 0.329<br>-2.683<br>**0.009**	-0.149 ± 0.329<br>-0.454<br>**0.652**	-0.721 ± 0.329<br>-2.192<br>**0.032**												
$J^{0.42}$ CV>HU=HD	0.394 ± 0.329<br>1.198<br>**0.236**	-0.705 ± 0.329<br>-2.143<br>**0.036**	0.028 ± 0.329<br>0.086<br>**0.932**	-0.544 ± 0.329<br>-1.653<br>**0.103**	0.178 ± 0.329<br>0.540<br>**0.591**											
$J^{0.64}$ HD>HU>CV	-1.531 ± 0.329<br>-4.655<br>**<0.0001**	-2.630 ± 0.329<br>-7.996<br>**<0.0001**	-1.897 ± 0.329<br>-5.767<br>**<0.0001**	-2.469 ± 0.329<br>-7.505<br>**<0.0001**	-1.747 ± 0.329<br>-5.313<br>**<0.0001**	-1.925 ± 0.329<br>-5.853<br>**<0.0001**										
$J^{0.92}$ HD>HU>CV	-1.107 ± 0.329<br>-3.365<br>**0.001**	-2.205 ± 0.329<br>-6.706<br>**<0.0001**	-1.472 ± 0.329<br>-4.476<br>**<0.0001**	-2.044 ± 0.329<br>-6.215<br>**<0.0001**	-1.323 ± 0.329<br>-4.023<br>**<0.0001**	-1.501 ± 0.329<br>-4.562<br>**<0.0001**	0.424 ± 0.329<br>1.290<br>**0.202**									
$J^{0.64}$ HD>CV>HU	-0.738 ± 0.329<br>-2.243<br>**0.028**	-1.837 ± 0.329<br>-5.584<br>**<0.0001**	-1.103 ± 0.329<br>-3.355<br>**0.001**	-1.675 ± 0.329<br>-5.094<br>**<0.0001**	-0.954 ± 0.329<br>-2.901<br>**0.005**	-1.132 ± 0.329<br>-3.441<br>**0.001**	0.793 ± 0.329<br>2.412<br>**0.019**	0.369 ± 0.329<br>1.122<br>**0.266**								
$J^{0.92}$ HD>CV>HU	-0.044 ± 0.329<br>-0.133<br>**0.895**	-1.143 ± 0.329<br>-3.474<br>**0.001**	-0.409 ± 0.329<br>-1.244<br>**0.218**	-0.981 ± 0.329<br>-2.983<br>**0.004**	-0.260 ± 0.329<br>-0.791<br>**0.432**	-0.438 ± 0.329<br>-1.330<br>**0.188**	1.487 ± 0.329<br>4.522<br>**<0.0001**	1.063 ± 0.329<br>3.232<br>**0.002**	0.694 ± 0.329<br>2.110<br>**0.039**							
$J^{0.42}$ HD>CV=HU	-1.469 ± 0.329<br>-4.467<br>**<0.0001**	-2.568 ± 0.329<br>-7.808<br>**<0.0001**	-1.835 ± 0.329<br>-5.579<br>**<0.0001**	-2.407 ± 0.329<br>-7.317<br>**<0.0001**	-1.686 ± 0.329<br>-5.125<br>**<0.0001**	-1.863 ± 0.329<br>-5.665<br>**<0.0001**	0.062 ± 0.329<br>0.188<br>**0.851**	-0.363 ± 0.329<br>-1.102<br>**0.274**	-0.731 ± 0.329<br>-2.224<br>**0.030**	-1.426 ± 0.329<br>-4.334<br>**<0.0001**						
$J^{0.64}$ HU>CV>HD	-1.022 ± 0.329<br>-3.108<br>**0.003**	-2.121 ± 0.329<br>-6.448<br>**<0.0001**	-1.388 ± 0.329<br>-4.219<br>**<0.0001**	-1.960 ± 0.329<br>-5.958<br>**<0.0001**	-1.238 ± 0.329<br>-3.765<br>**<0.0001**	-1.416 ± 0.329<br>-4.305<br>**<0.0001**	0.509 ± 0.329<br>1.548<br>**0.127**	0.085 ± 0.329<br>0.257<br>**0.798**	-0.284 ± 0.329<br>-0.864<br>**0.391**	-0.978 ± 0.329<br>-2.975<br>**0.004**	0.447 ± 0.329<br>1.360<br>**0.179**					
$J^{0.92}$ HU>CV>HD	-0.071 ± 0.329<br>-0.215<br>**0.830**	-1.170 ± 0.329<br>-3.556<br>**0.001**	-0.436 ± 0.329<br>-1.327<br>**0.189**	-1.008 ± 0.329<br>-3.065<br>**0.003**	-0.287 ± 0.329<br>-0.873<br>**0.386**	-0.465 ± 0.329<br>-1.413<br>**0.163**	1.460 ± 0.329<br>4.440<br>**<0.0001**	1.036 ± 0.329<br>3.150<br>**0.002**	0.667 ± 0.329<br>2.028<br>**0.047**	-0.027 ± 0.329<br>-0.082<br>**0.935**	1.398 ± 0.329<br>4.252<br>**<0.0001**	0.951 ± 0.329<br>2.892<br>**0.005**				
$J^{0.64}$ HU>HD>CV	-1.524 ± 0.329<br>-4.634<br>**<0.0001**	-2.623 ± 0.329<br>-7.975<br>**<0.0001**	-1.890 ± 0.329<br>-5.746<br>**<0.0001**	-2.462 ± 0.329<br>-7.484<br>**<0.0001**	-1.741 ± 0.329<br>-5.292<br>**<0.0001**	-1.918 ± 0.329<br>-5.832<br>**<0.0001**	0.007 ± 0.329<br>0.021<br>**0.983**	-0.418 ± 0.329<br>-1.269<br>**0.209**	-0.786 ± 0.329<br>-2.391<br>**0.020**	-1.480 ± 0.329<br>-4.501<br>**<0.0001**	-0.055 ± 0.329<br>-0.167<br>**0.868**	-0.502 ± 0.329<br>-1.527<br>**0.132**	-1.453 ± 0.329<br>-4.419<br>**<0.0001**			
$J^{0.92}$ HU>HD>CV	-0.848 ± 0.329<br>-2.579<br>**0.012**	-1.947 ± 0.329<br>-5.920<br>**<0.0001**	-1.214 ± 0.329<br>-3.691<br>**<0.0001**	-1.786 ± 0.329<br>-5.429<br>**<0.0001**	-1.065 ± 0.329<br>-3.237<br>**0.002**	-1.242 ± 0.329<br>-3.777<br>**<0.0001**	0.683 ± 0.329<br>2.076<br>**0.042**	0.258 ± 0.329<br>0.786<br>**0.435**	-0.110 ± 0.329<br>-0.336<br>**0.738**	-0.805 ± 0.329<br>-2.446<br>**0.017**	0.621 ± 0.329<br>1.888<br>**0.064**	0.174 ± 0.329<br>0.529<br>**0.599**	-0.777 ± 0.329<br>-2.364<br>**0.021**	0.676 ± 0.329<br>2.055<br>**0.044**		
$J^{0.42}$ HU>HD=CV	-1.677 ± 0.329<br>-5.098<br>**<0.0001**	-2.776 ± 0.329<br>-8.439<br>**<0.0001**	-2.042 ± 0.329<br>-6.210<br>**<0.0001**	-2.614 ± 0.329<br>-7.948<br>**<0.0001**	-1.893 ± 0.329<br>-5.756<br>**<0.0001**	-2.071 ± 0.329<br>-6.296<br>**<0.0001**	-0.146 ± 0.329<br>-0.443<br>**0.659**	-0.570 ± 0.329<br>-1.733<br>**0.088**	-0.939 ± 0.329<br>-2.855<br>**0.006**	-1.633 ± 0.329<br>-4.965<br>**<0.0001**	-0.208 ± 0.329<br>-0.631<br>**0.530**	-0.655 ± 0.329<br>-1.990<br>**0.051**	-1.606 ± 0.329<br>-4.883<br>**<0.0001**	-0.153 ± 0.329<br>-0.464<br>**0.644**	-0.829 ± 0.329<br>-2.519<br>**0.014**	

Supplementary model S19: Surface boundary roughness (SBR, cm)

Initial linear regression model:

Lm(SBR~ SpD)

Minimal adequate model:

gls(SBR~ SpD, weights = varIdent (form = ~ 1|SpD), method = 'ML')

(SBR, L-ratio = 36.98, d.f. = 15, p = 0.001)

Coefficient table model S20 (method = 'REML'): Intercept ± SE (For baseline J = 1): 0.927 ± 0.162, t = 5.717, p = <0.0001. Coefficients ± SE and t-values are presented. Significance values are in bold. CV = *Corophium volutator*, HD = *Hediste diversicolor*, HU = *Hydrobia ulvae*

	$J^{1.00}$	$J^{0.64}$ CV>HD>HU	$J^{0.92}$ CV>HD>HU	$J^{0.64}$ CV>HU>HD	$J^{0.92}$ CV>HU>HD	$J^{0.42}$ CV>HU=HD	$J^{0.64}$ HD>HU>CV	$J^{0.92}$ HD>HU>CV	$J^{0.64}$ HD>CV>HU	$J^{0.92}$ HD>CV>HU	$J^{0.42}$ HD>CV=HU	$J^{0.64}$ HU>CV>HD	$J^{0.92}$ HU>CV>HD	$J^{0.64}$ HU>HD>CV	$J^{0.92}$ HU>HD>CV
$J^{0.64}$ CV>HD>HU	0.022 ± 0.196<br>0.114<br>**0.910**														
$J^{0.92}$ CV>HD>HU	0.0528 ± 0.201<br>0.262<br>**0.794**	0.0305 ± 0.162<br>0.188<br>**0.851**													
$J^{0.64}$ CV>HU>HD	0.327 ± 0.230<br>1.423<br>**0.160**	0.304 ± 0.196<br>1.554<br>**0.125**	0.282 ± 0.142<br>1.983<br>**0.052**												
$J^{0.92}$ CV>HU>HD	0.205 ± 0.194<br>1.057<br>**0.294**	0.183 ± 0.152<br>1.198<br>**0.235**	0.555 ± 0.180<br>3.091<br>**0.003**	-0.274 ± 0.202<br>-1.358<br>**0.179**											
$J^{0.42}$ CV>HU=HD	0.238 ± 0.2814<br>0.847<br>**0.400**	0.216 ± 0.255<br>0.848<br>**0.400**	0.434 ± 0.131<br>3.314<br>**0.002**	-0.122 ± 0.194<br>-0.628<br>**0.532**	-0.152 ± 0.160<br>-0.952<br>**0.344**										
$J^{0.64}$ HD>HU>CV	-0.112 ± 0.182<br>-0.616<br>**0.540**	-0.135 ± 0.137<br>-0.979<br>**0.331**	0.467 ± 0.242<br>1.927<br>**0.058**	-0.088 ± 0.281<br>-0.314<br>**0.755**	0.034 ± 0.253<br>0.133<br>**0.895**	-0.351 ± 0.244<br>-1.434<br>**0.156**									
$J^{0.92}$ HD>HU>CV	-0.082 ± 0.171<br>-0.478<br>**0.634**	-0.104 ± 0.122<br>-0.853<br>**0.397**	0.117 ± 0.113<br>1.031<br>**0.307**	-0.439 ± 0.182<br>-2.406<br>**0.019**	-0.317 ± 0.134<br>-2.357<br>**0.022**	-0.320 ± 0.236<br>-1.356<br>**0.180**	0.031 ± 0.098<br>0.312<br>**0.756**								
$J^{0.64}$ HD>CV>HU	-0.077 ± 0.168<br>-0.458<br>**0.648**	-0.099 ± 0.118<br>-0.841<br>**0.403**	0.147 ± 0.093<br>1.578<br>**0.120**	-0.408 ± 0.171<br>-2.389<br>**0.020**	-0.286 ± 0.118<br>-2.419<br>**0.018**	-0.315 ± 0.234<br>-1.347<br>**0.183**	0.035 ± 0.094<br>0.377<br>**0.707**	0.005 ± 0.069<br>0.068<br>**0.946**							
$J^{0.92}$ HD>CV>HU	-0.229 ± 0.179<br>-1.275<br>**0.207**	-0.251 ± 0.134<br>-1.876<br>**0.065**	0.152 ± 0.088<br>1.719<br>**0.090**	-0.403 ± 0.168<br>-2.400<br>**0.019**	-0.282 ± 0.114<br>-2.461<br>**0.017**	-0.467 ± 0.242<br>-1.927<br>**0.058**	-0.117 ± 0.113<br>-1.031<br>**0.307**	-0.147 ± 0.093<br>-1.578<br>**0.120**	-0.152 ± 0.088<br>-1.719<br>**0.090**						
$J^{0.42}$ HD>CV=HU	-0.115 ± 0.181<br>-0.637<br>**0.526**	-0.137 ± 0.135<br>-1.015<br>**0.314**	0.114 ± 0.110<br>1.031<br>**0.307**	-0.442 ± 0.181<br>-2.443<br>**0.017**	-0.320 ± 0.132<br>-2.418<br>**0.019**	-0.353 ± 0.243<br>-1.454<br>**0.151**	-0.003 ± 0.115<br>-0.024<br>**0.981**	-0.033 ± 0.095<br>-0.351<br>**0.727**	-0.038 ± 0.090<br>-0.421<br>**0.675**	0.114 ± 0.110<br>1.031<br>**0.307**					
$J^{0.64}$ HU>CV>HD	-0.155 ± 0.193<br>-0.805<br>**0.424**	-0.178 ± 0.152<br>-1.172<br>**0.246**	0.073 ± 0.130<br>0.565<br>**0.574**	-0.482 ± 0.193<br>-2.493<br>**0.015**	-0.360 ± 0.149<br>-2.418<br>**0.019**	-0.394 ± 0.253<br>-1.558<br>**0.124**	-0.043 ± 0.134<br>-0.322<br>**0.748**	-0.074 ± 0.117<br>-0.629<br>**0.532**	-0.078 ± 0.113<br>-0.691<br>**0.492**	0.073 ± 0.130<br>0.565<br>**0.574**	-0.040 ± 0.131<br>-0.307<br>**0.760**				
$J^{0.92}$ HU>CV>HD	0.020 ± 0.237<br>0.084534<br>**0.933**	-0.002 ± 0.204<br>-0.011<br>**0.991**	0.249 ± 0.189<br>1.317<br>**0.193**	-0.307 ± 0.237<br>-1.293<br>**0.201**	-0.185 ± 0.203<br>-0.912<br>**0.365**	-0.218 ± 0.288<br>-0.759<br>**0.451**	0.132 ± 0.192<br>0.691<br>**0.492**	0.102 ± 0.181<br>0.562<br>**0.576**	0.097 ± 0.178<br>0.544<br>**0.588**	0.249 ± 0.189<br>1.317<br>**0.193**	0.135 ± 0.190<br>0.711<br>**0.480**	0.175 ± 0.202<br>0.868<br>**0.389**			
$J^{0.64}$ HU>HD>CV	-0.222 ± 0.167<br>-1.324<br>**0.190**	-0.244 ± 0.117<br>-2.082<br>**0.041**	0.007 ± 0.087<br>0.083<br>**0.934**	-0.548 ± 0.168<br>-3.270<br>**0.002**	-0.426 ± 0.114<br>-3.750<br>**<0.001**	-0.460 ± 0.234<br>-1.968<br>**0.053**	-0.109 ± 0.093<br>-1.178<br>**0.243**	-0.140 ± 0.067<br>-2.082<br>**0.041**	-0.145 ± 0.060<br>-2.405<br>**0.019**	0.007 ± 0.087<br>0.083<br>**0.934**	-0.107 ± 0.089<br>-1.191<br>**0.238**	-0.066 ± 0.113<br>-0.587<br>**0.559**	-0.242 ± 0.178<br>-1.360<br>**0.179**		
$J^{0.92}$ HU>HD>CV	-0.284 ± 0.178<br>-1.593<br>**0.116**	-0.306 ± 0.132<br>-2.315<br>**0.024**	-0.055 ± 0.107<br>-0.516<br>**0.607**	-0.611 ± 0.179<br>-3.420<br>**0.001**	-0.489 ± 0.129<br>-3.782<br>**<0.001**	-0.522 ± 0.242<br>-2.162<br>**0.034**	-0.172 ± 0.111<br>-1.544<br>**0.128**	-0.202 ± 0.091<br>-2.222<br>**0.030**	-0.207 ± 0.086<br>-2.409<br>**0.019**	-0.055 ± 0.107<br>-0.516<br>**0.607**	-0.169 ± 0.109<br>-1.557<br>**0.124**	-0.129 ± 0.128<br>-1.002<br>**0.320**	-0.304 ± 0.188<br>-1.618<br>**0.111**	-0.062 ± 0.085<br>-0.735<br>**0.465**	
$J^{0.42}$ HU>HD=CV	-0.244 ± 0.168<br>-1.452<br>**0.152**	-0.266 ± 0.118<br>-2.257<br>**0.027**	-0.015 ± 0.088<br>-0.169<br>**0.866**	-0.570 ± 0.168<br>-3.392<br>**0.001**	-0.449 ± 0.114<br>-3.918<br>**<0.001**	-0.482 ± 0.234<br>-2.060<br>**0.044**	-0.132 ± 0.094<br>-1.404<br>**0.165**	-0.162 ± 0.069<br>-2.366<br>**0.021**	-0.167 ± 0.062<br>-2.709<br>**0.009**	-0.015 ± 0.088<br>-0.169<br>**0.866**	-0.129 ± 0.090<br>-1.424<br>**0.159**	-0.088 ± 0.114<br>-0.779<br>**0.439**	-0.264 ± 0.178<br>-1.481<br>**0.143**	-0.022 ± 0.060<br>-0.369<br>**0.713**	0.040 ± 0.086<br>0.468<br>**0.642**

$J^{0.42}$ HU>HD=CV

Supplementary model S20: Max mixed depth of particle reworking ($^{f\text{-}SPI}L_{max}$, cm)

Initial linear regression model:

Lm($^{f\text{-}SPI}L_{max}$~ SpD)

No minimal adequate model, intercept only (SpD, $F = 1.29$, d.f. = 15, $p = 0.237$)

Supplementary model S21: Bioirrigation ($\Delta[Br^-]$, mg L^{-1})

Initial linear regression model:

Lm($\Delta[Br^-]$ ~ SpD)

Minimal adequate model:

gls($\Delta[Br^-]$ ~ SpD, weights = varIdent (form = ~ 1|SpD), method = 'ML')

(SpD, L-ratio = 26.06, d.f. = 15, $p = 0.037$)

Coefficient table model S21 (method = 'REML'): Intercept ± SE (For baseline J = 1): -65.983 ± 49.668, t = -1.328, p = 0.189. Coefficients ± SE and t-values are presented. Significance values are in bold. CV = *Corophium volutator*, HD = *Hediste diversicolor*, HU = *Hydrobia ulvae*

	$J^{1.00}$	$J^{0.64}$ CV>HD>HU	$J^{0.92}$ CV>HD>HU	$J^{0.64}$ CV>HU>HD	$J^{0.92}$ CV>HU>HD	$J^{0.42}$ CV>HU=HD	$J^{0.64}$ HD>HU>CV	$J^{0.92}$ HD>HU>CV	$J^{0.64}$ HD>CV>HU	$J^{0.92}$ HD>CV>HU	$J^{0.42}$ HD>CV=HU	$J^{0.64}$ HU>CV>HD	$J^{0.92}$ HU>CV>HD	$J^{0.64}$ HU>HD>CV	$J^{0.92}$ HU>HD>CV	$J^{0.42}$ HU>HD=CV
$J^{0.64}$ CV>HD>HU	13.900 ± 62.673<br>0.222<br>**0.825**															
$J^{0.92}$ CV>HD>HU	-60.752 ± 109.759<br>-0.554<br>**0.582**	-74.653 ± 105.077<br>-0.710<br>**0.480**														
$J^{0.64}$ CV>HU>HD	124.089 ± 50.416<br>2.461<br>**0.017**	110.189 ± 39.189<br>2.812<br>**0.007**	184.841 ± 98.260<br>1.881<br>**0.065**													
$J^{0.92}$ CV>HU>HD	133.081 ± 101.972<br>1.305<br>**0.197**	119.181 ± 96.913<br>1.230<br>**0.223**	193.833 ± 132.331<br>1.465<br>**0.148**	8.992 ± 89.477<br>0.100<br>**0.920**												
$J^{0.42}$ CV>HU=HD	64.734 ± 73.867<br>0.876<br>**0.384**	50.833 ± 66.711<br>0.762<br>**0.449**	125.486 ± 112.114<br>1.119<br>**0.267**	-59.355 ± 55.356<br>-1.072<br>**0.288**	-68.348 ± 104.502<br>-0.654<br>**0.515**											
$J^{0.64}$ HD>HU>CV	-30.806 ± 103.813<br>-0.297<br>**0.768**	-44.706 ± 98.849<br>-0.452<br>**0.653**	29.947 ± 133.755<br>0.224<br>**0.824**	-154.895 ± 91.570<br>-1.692<br>**0.096**	-163.887 ± 127.442<br>-1.286<br>**0.203**	-95.539 ± 106.299<br>-0.899<br>**0.372**										
$J^{0.92}$ HD>HU>CV	92.826 ± 54.593<br>1.700<br>**0.094**	78.925 ± 44.435<br>1.776<br>**0.081**	153.578 ± 100.468<br>1.529<br>**0.131**	-31.263 ± 24.257<br>-1.289<br>**0.202**	-40.256 ± 91.896<br>-0.438<br>**0.663**	28.092 ± 59.185<br>0.475<br>**0.637**	123.631 ± 93.934<br>1.316<br>**0.193**									
$J^{0.64}$ HD>CV>HU	1.162 ± 98.178<br>0.012<br>**0.991**	-12.739 ± 92.914<br>-0.137<br>**0.891**	61.914 ± 129.431<br>0.478<br>**0.634**	-122.927 ± 85.129<br>-1.444<br>**0.154**	-131.919 ± 122.896<br>-1.073<br>**0.287**	-63.572 ± 100.804<br>-0.631<br>**0.531**	31.967 ± 124.428<br>0.257<br>**0.798**	-91.664 ± 87.667<br>-1.046<br>**0.300**								
$J^{0.92}$ HD>CV>HU	71.115 ± 75.006<br>0.948<br>**0.347**	57.215 ± 67.970<br>0.842<br>**0.403**	131.867 ± 112.868<br>1.168<br>**0.247**	-52.974 ± 56.867<br>-0.932<br>**0.355**	-61.966 ± 105.310<br>-0.588<br>**0.558**	6.382 ± 78.411<br>0.081<br>**0.935**	101.921 ± 107.094<br>0.952<br>**0.345**	-21.710 ± 60.601<br>-0.358<br>**0.721**	69.953 ± 101.642<br>0.688<br>**0.494**							
$J^{0.42}$ HD>CV=HU	61.282 ± 51.781<br>1.183<br>**0.241**	47.381 ± 40.930<br>1.158<br>**0.251**	122.034 ± 98.967<br>1.233<br>**0.222**	-62.807 ± 17.006<br>-3.693<br>**0.001**	-71.800 ± 90.253<br>-0.796<br>**0.429**	-3.452 ± 56.601<br>-0.061<br>**0.952**	92.087 ± 92.328<br>0.997<br>**0.322**	-31.544 ± 26.979<br>-1.169<br>**0.247**	60.120 ± 85.944<br>0.700<br>**0.487**	-9.834 ± 58.080<br>-0.169<br>**0.866**						
$J^{0.64}$ HU>CV>HD	5.185 ± 101.491<br>0.051<br>**0.959**	-8.716 ± 96.408<br>-0.090<br>**0.928**	65.937 ± 131.961<br>0.500<br>**0.619**	-118.904 ± 88.929<br>-1.337<br>**0.186**	-127.897 ± 125.558<br>-1.019<br>**0.312**	-59.549 ± 104.033<br>-0.572<br>**0.569**	35.990 ± 127.058<br>0.283<br>**0.778**	-87.641 ± 91.362<br>-0.959<br>**0.341**	4.023 ± 122.497<br>0.033<br>**0.974**	-65.931 ± 104.845<br>-0.629<br>**0.532**	-56.097 ± 89.710<br>-0.625<br>**0.534**					
$J^{0.92}$ HU>CV>HD	36.293 ± 96.090<br>0.378<br>**0.707**	22.393 ± 90.704<br>0.247<br>**0.806**	97.045 ± 127.854<br>0.759<br>**0.451**	-87.796 ± 82.711<br>-1.061<br>**0.293**	-96.788 ± 121.234<br>-0.798<br>**0.428**	-28.440 ± 98.771<br>-0.288<br>**0.774**	67.099 ± 122.786<br>0.546<br>**0.587**	-56.532 ± 85.322<br>-0.663<br>**0.510**	35.131 ± 118.061<br>0.298<br>**0.767**	-34.822 ± 99.626<br>-0.350<br>**0.728**	-24.988 ± 83.550<br>-0.299<br>**0.766**	31.109 ± 120.830<br>0.257<br>**0.798**				
$J^{0.64}$ HU>HD>CV	42.508 ± 57.731<br>0.736<br>**0.464**	28.607 ± 48.238<br>0.593<br>**0.555**	103.260 ± 102.207<br>1.010<br>**0.316**	-81.581 ± 30.674<br>-2.660<br>**0.010**	-90.573 ± 93.794<br>-0.966<br>**0.338**	-22.226 ± 62.092<br>-0.358<br>**0.722**	73.313 ± 95.792<br>0.765<br>**0.447**	-50.318 ± 37.142<br>-1.355<br>**0.180**	41.346 ± 89.655<br>0.461<br>**0.646**	-28.607 ± 63.443<br>-0.451<br>**0.654**	-18.774 ± 32.868<br>-0.571<br>**0.570**	37.323 ± 93.271<br>0.400<br>**0.690**	6.215 ± 87.363<br>0.071<br>**0.944**			
$J^{0.92}$ HU>HD>CV	-44.838 ± 106.727<br>-0.420<br>**0.676**	-58.739 ± 101.905<br>-0.576<br>**0.566**	15.914 ± 136.029<br>0.117<br>**0.907**	-168.927 ± 94.861<br>-1.781<br>**0.080**	-177.919 ± 129.827<br>-1.370<br>**0.175**	-109.572 ± 109.147<br>-1.004<br>**0.319**	-14.033 ± 131.278<br>-0.107<br>**0.915**	-137.664 ± 97.146<br>-1.417<br>**0.161**	-46.000 ± 126.869<br>-0.363<br>**0.718**	-115.953 ± 109.922<br>-1.055<br>**0.295**	-106.120 ± 95.593<br>-1.110<br>**0.271**	-50.023 ± 129.450<br>-0.386<br>**0.701**	-81.131 ± 125.260<br>-0.648<br>**0.520**	-87.346 ± 98.943<br>-0.883<br>**0.381**		
$J^{0.42}$ HU>HD=CV	42.377 ± 52.894<br>0.801<br>**0.426**	28.477 ± 42.330<br>0.673<br>**0.504**	103.130 ± 99.554<br>1.036<br>**0.304**	-81.712 ± 20.143<br>-4.057<br>**<0.0001**	-90.704 ± 90.896<br>-0.998<br>**0.322**	-22.356 ± 57.621<br>-0.388<br>**0.699**	73.183 ± 92.957<br>0.787<br>**0.434**	-50.448 ± 29.058<br>-1.736<br>**0.087**	41.216 ± 86.619<br>0.476<br>**0.636**	-28.738 ± 59.075<br>-0.486<br>**0.628**	-18.904 ± 23.349<br>-0.810<br>**0.421**	37.193 ± 90.357<br>0.412<br>**0.682**	6.084 ± 84.245<br>0.072<br>**0.943**	-0.130 ± 34.596<br>-0.004<br>**0.997**	87.216 ± 96.201<br>0.907<br>**0.368**	

Supplementary model S22: NH_4-N concentration ([NH_4-N], mg L^{-1})

Initial linear regression model:

Lm([NH_4-N] ~ SpD)

Minimal adequate model:

gls([NH_4-N] ~ SpD, weights = varIdent (form = ~ 1|SpD), method = 'ML')

(SpD, L-ratio = 79.21, d.f. = 15, p = <0.0001)

Coefficient table model S23 (method = 'REML'): Intercept ± SE (For baseline J = 1): 2.097 ± 0.258, t = 8.140, p < 0.0001. Coefficients ± SE and t-values are presented. Significance values are in bold. CV = *Corophium volutator*, HD = *Hediste diversicolor*, HU = *Hydrobia ulvae*

	$J^{1.00}$	$J^{0.64}$ CV>HD>HU	$J^{0.92}$ CV>HD>HU	$J^{0.64}$ CV>HU>HD	$J^{0.92}$ CV>HU>HD	$J^{0.42}$ CV>HU=HD	$J^{0.64}$ HD>HU>CV	$J^{0.92}$ HD>HU>CV	$J^{0.64}$ HD>CV>HU	$J^{0.92}$ HD>CV>HU	$J^{0.42}$ HD>CV=HU	$J^{0.64}$ HU>CV>HD	$J^{0.92}$ HU>CV>HD	$J^{0.64}$ HU>HD>CV	$J^{0.92}$ HU>HD>CV	$J^{0.42}$ HU>HD=CV
$J^{0.64}$ CV>HD>HU	-1.275 ± 0.374<br>-3.406<br>**0.001**															
$J^{0.92}$ CV>HD>HU	-0.740 ± 0.373<br>-1.985<br>**0.052**	0.535 ± 0.382<br>1.398<br>**0.167**														
$J^{0.64}$ CV>HU>HD	-1.127 ± 0.351<br>-3.212<br>**0.002**	0.148 ± 0.361<br>0.408<br>**0.685**	-0.387 ± 0.360<br>-1.076<br>**0.286**													
$J^{0.92}$ CV>HU>HD	-0.161 ± 0.305<br>-0.528<br>**0.600**	1.114 ± 0.317<br>3.513<br>**0.001**	0.579 ± 0.315<br>1.836<br>**0.071**	0.966 ± 0.289<br>3.340<br>**0.002**												
$J^{0.42}$ CV>HU=HD	-0.504 ± 0.634<br>-0.795<br>**0.430**	0.771 ± 0.640<br>1.205<br>**0.233**	0.236 ± 0.639<br>0.370<br>**0.713**	0.623 ± 0.626<br>0.995<br>**0.324**	-0.343 ± 0.602<br>-0.570<br>**0.571**											
$J^{0.64}$ HD>HU>CV	0.755 ± 0.282<br>2.680<br>**0.010**	2.030 ± 0.294<br>6.895<br>**<0.0001**	1.495 ± 0.292<br>5.111<br>**<0.0001**	1.882 ± 0.264<br>7.124<br>**<0.0001**	0.916 ± 0.199<br>4.593<br>**<0.0001**	1.259 ± 0.590<br>2.133<br>**0.037**										
$J^{0.92}$ HD>HU>CV	0.344 ± 0.325<br>1.056<br>**0.295**	1.618 ± 0.336<br>4.811<br>**<0.0001**	1.084 ± 0.335<br>3.237<br>**0.002**	1.471 ± 0.310<br>4.739<br>**<0.0001**	0.505 ± 0.257<br>1.960<br>**0.055**	0.848 ± 0.612<br>1.384<br>**0.172**	-0.411 ± 0.229<br>-1.796<br>**0.078**									
$J^{0.64}$ HD>CV>HU	0.615 ± 0.329<br>1.870<br>**0.067**	1.890 ± 0.340<br>5.558<br>**<0.0001**	1.355 ± 0.338<br>4.005<br>**<0.0001**	1.743 ± 0.314<br>5.544<br>**<0.0001**	0.776 ± 0.262<br>2.961<br>**0.004**	1.119 ± 0.614<br>1.822<br>**0.074**	-0.140 ± 0.234<br>-0.596<br>**0.554**	0.272 ± 0.285<br>0.952<br>**0.345**								
$J^{0.92}$ HD>CV>HU	0.056 ± 0.341<br>0.165<br>**0.869**	1.331 ± 0.352<br>3.783<br>**<0.0001**	0.796 ± 0.350<br>2.274<br>**0.027**	1.184 ± 0.327<br>3.619<br>**0.001**	0.217 ± 0.277<br>0.784<br>**0.436**	0.560 ± 0.621<br>0.903<br>**0.370**	-0.698 ± 0.251<br>-2.781<br>**0.007**	-0.287 ± 0.299<br>-0.960<br>**0.341**	-0.559 ± 0.303<br>-1.842<br>**0.071**							
$J^{0.42}$ HD>CV=HU	0.924 ± 0.316<br>2.924<br>**0.005**	2.199 ± 0.327<br>6.715<br>**<0.0001**	1.664 ± 0.326<br>5.108<br>**<0.0001**	2.051 ± 0.301<br>6.823<br>**<0.0001**	1.085 ± 0.246<br>4.417<br>**<0.0001**	1.428 ± 0.607<br>2.351<br>**0.022**	0.169 ± 0.216<br>0.785<br>**0.435**	0.580 ± 0.270<br>2.148<br>**0.036**	0.309 ± 0.275<br>1.124<br>**0.266**	0.868 ± 0.289<br>3.000<br>**0.004**						
$J^{0.64}$ HU>CV>HD	-0.267 ± 0.277<br>-0.966<br>**0.338**	1.008 ± 0.290<br>3.480<br>**0.001**	0.473 ± 0.288<br>1.644<br>**0.106**	0.860 ± 0.259<br>3.323<br>**0.002**	-0.106 ± 0.192<br>-0.552<br>**0.583**	0.237 ± 0.588<br>0.403<br>**0.689**	-1.022 ± 0.152<br>-6.728<br>**<0.0001**	-0.611 ± 0.223<br>-2.743<br>**0.008**	-0.882 ± 0.228<br>-3.868<br>**<0.0001**	-0.324 ± 0.245<br>-1.319<br>**0.193**	-1.191 ± 0.209<br>-5.701<br>**<0.0001**					
$J^{0.92}$ HU>CV>HD	-0.901 ± 0.278<br>-3.245<br>**0.002**	0.374 ± 0.291<br>1.286<br>**0.204**	-0.161 ± 0.289<br>-0.558<br>**0.579**	0.226 ± 0.260<br>0.870<br>**0.388**	-0.740 ± 0.194<br>-3.817<br>**<0.0001**	-0.397 ± 0.588<br>-0.675<br>**0.502**	-1.656 ± 0.154<br>-10.754<br>**<0.0001**	-1.245 ± 0.224<br>-5.553<br>**<0.0001**	-1.516 ± 0.230<br>-6.605<br>**<0.0001**	-0.957 ± 0.247<br>-3.881<br>**<0.0001**	-1.825 ± 0.210<br>-8.672<br>**<0.0001**	-0.634 ± 0.145<br>-4.385<br>**<0.0001**				
$J^{0.64}$ HU>HD>CV	-0.036 ± 0.271<br>-0.134<br>**0.894**	1.238 ± 0.284<br>4.356<br>**<0.0001**	0.704 ± 0.282<br>2.492<br>**0.016**	1.091 ± 0.253<br>4.313<br>**<0.0001**	0.125 ± 0.184<br>0.677<br>**0.501**	0.468 ± 0.585<br>0.799<br>**0.428**	-0.791 ± 0.142<br>-5.587<br>**<0.0001**	-0.380 ± 0.216<br>-1.761<br>**0.084**	-0.652 ± 0.221<br>-2.943<br>**0.005**	-0.093 ± 0.239<br>-0.388<br>**0.700**	-0.960 ± 0.202<br>-4.764<br>**<0.0001**	0.231 ± 0.131<br>1.758<br>**0.084**	0.865 ± 0.134<br>6.467<br>**<0.0001**			
$J^{0.92}$ HU>HD>CV	0.239 ± 0.276<br>0.864<br>**0.391**	1.513 ± 0.289<br>5.232<br>**<0.0001**	0.979 ± 0.287<br>3.406<br>**0.001**	1.366 ± 0.259<br>5.284<br>**<0.0001**	0.400 ± 0.192<br>2.083<br>**0.042**	0.743 ± 0.588<br>1.264<br>**0.211**	-0.516 ± 0.151<br>-3.409<br>**0.001**	-0.105 ± 0.222<br>-0.472<br>**0.639**	-0.377 ± 0.228<br>-1.653<br>**0.104**	0.182 ± 0.245<br>0.743<br>**0.460**	-0.685 ± 0.209<br>-3.286<br>**0.002**	0.506 ± 0.142<br>3.567<br>**0.001**	1.140 ± 0.144<br>7.913<br>**<0.0001**	0.275 ± 0.131<br>2.104<br>**0.040**		
$J^{0.42}$ HU>HD=CV	-0.274 ± 0.310<br>-0.882<br>**0.381**	1.001 ± 0.322<br>3.112<br>**0.003**	0.467 ± 0.320<br>1.458<br>**0.150**	0.854 ± 0.294<br>2.900<br>**0.005**	-0.113 ± 0.238<br>-0.473<br>**0.638**	0.230 ± 0.604<br>0.381<br>**0.704**	-1.028 ± 0.207<br>-4.973<br>**<0.0001**	-0.617 ± 0.263<br>-2.344<br>**0.023**	-0.889 ± 0.268<br>-3.318<br>**0.002**	-0.330 ± 0.283<br>-1.167<br>**0.248**	-1.198 ± 0.252<br>-4.758<br>**<0.0001**	-0.006 ± 0.200<br>-0.032<br>**0.975**	0.628 ± 0.201<br>3.115<br>**0.003**	-0.237 ± 0.192<br>-1.234<br>**0.222**	-0.512 ± 0.199<br>-2.567<br>**0.013**	

Supplementary model S23: NO_X-N concentration ([NO_X-N], mg L^{-1})

Initial linear regression model:

Lm([NO_X-N] ~ SpD)

Minimal adequate model:

gls([NO_X-N] ~ SpD, weights = varIdent (form = ~ 1|SpD), method = 'ML')

(SpD, L-ratio = 8.53, d.f. = 15, p = <0.0001)

Coefficient table model S22: Intercept ± SE (For baseline J = 1): 0.556 ± 0.064, t = 8.696, p = <0.0001. Coefficients ± SE and t-values are presented. Significance values are in bold. CV = *Corophium volutator*, HD = *Hediste diversicolor*, HU = *Hydrobia ulvae*

	$J^{1.00}$	$J^{0.64}$ CV>HD>HU	$J^{0.92}$ CV>HD>HU	$J^{0.64}$ CV>HU>HD	$J^{0.92}$ CV>HU>HD	$J^{0.42}$ CV>HU=HD	$J^{0.64}$ HD>HU>CV	$J^{0.92}$ HD>HU>CV	$J^{0.64}$ HD>CV>HU	$J^{0.92}$ HD>CV>HU	$J^{0.42}$ HD>CV=HU	$J^{0.64}$ HU>CV>HD	$J^{0.92}$ HU>CV>HD	$J^{0.64}$ HU>HD>CV	$J^{0.92}$ HU>HD>CV
$J^{0.64}$ CV>HD>HU	0.522 ± 0.090<br>5.782<br>**<0.0001**														
$J^{0.92}$ CV>HD>HU	0.237 ± 0.096<br>2.459<br>**0.017**	-0.287 ± 0.096<br>-2.992<br>**0.004**													
$J^{0.64}$ CV>HU>HD	0.506 ± 0.096<br>5.282<br>**<0.0001**	-0.016 ± 0.096<br>-0.170<br>**0.866**	0.271 ± 0.101<br>2.678<br>**0.010**												
$J^{0.92}$ CV>HU>HD	0.130 ± 0.096<br>1.355<br>**0.181**	-0.393 ± 0.096<br>-4.096<br>**0.0001**	-0.106 ± 0.101<br>-1.047<br>**0.300**	-0.376 ± 0.101<br>-3.725<br>**<0.001**											
$J^{0.42}$ CV>HU=HD	0.464 ± 0.090<br>5.131<br>**<0.0001**	-0.059 ± 0.090<br>-0.651<br>**0.518**	0.228 ± 0.096<br>2.379<br>**0.021**	-0.043 ± 0.096<br>-0.444<br>**0.659**	0.334 ± 0.096<br>3.482<br>**0.001**										
$J^{0.64}$ HD>HU>CV	0.049 ± 0.096<br>0.513<br>**0.610**	-0.473 ± 0.096<br>-4.938<br>**<0.0001**	-0.187 ± 0.101<br>-1.846<br>**0.070**	-0.457 ± 0.101<br>-4.524<br>**<0.0001**	-0.081 ± 0.101<br>-0.799<br>**0.427**	-0.414 ± 0.096<br>-4.325<br>**0.0001**									
$J^{0.92}$ HD>HU>CV	0.083 ± 0.090<br>0.923<br>**0.360**	-0.439 ± 0.090<br>-4.859<br>**<0.0001**	-0.152 ± 0.096<br>-1.589<br>**0.118**	-0.423 ± 0.096<br>-4.411<br>**<0.0001**	-0.047 ± 0.096<br>-0.485<br>**0.629**	-0.380 ± 0.090<br>-4.208<br>**0.0001**	0.034 ± 0.096<br>0.357<br>**0.722**								
$J^{0.64}$ HD>CV>HU	-0.068 ± 0.090<br>-0.757<br>**0.452**	-0.591 ± 0.090<br>-6.539<br>**<0.0001**	-0.304 ± 0.096<br>-3.173<br>**0.002**	-0.575 ± 0.096<br>-5.995<br>**<0.0001**	-0.198 ± 0.096<br>-2.069<br>**0.043**	-0.532 ± 0.090<br>-5.888<br>**<0.0001**	-0.118 ± 0.096<br>-1.227<br>**0.225**	-0.152 ± 0.090<br>-1.680<br>**0.098**							
$J^{0.92}$ HD>CV>HU	-0.036 ± 0.090<br>-0.398<br>**0.692**	-0.558 ± 0.090<br>-6.180<br>**<0.0001**	-0.272 ± 0.096<br>-2.835<br>**0.006**	-0.542 ± 0.096<br>-5.657<br>**<0.0001**	-0.166 ± 0.096<br>-1.731<br>**0.089**	-0.500 ± 0.090<br>-5.529<br>**<0.0001**	-0.085 ± 0.096<br>-0.889<br>**0.378**	-0.119 ± 0.090<br>-1.321<br>**0.192**	0.032 ± 0.090<br>0.359<br>**0.721**						
$J^{0.42}$ HD>CV=HU	-0.047 ± 0.090<br>-0.522<br>**0.603**	-0.570 ± 0.090<br>-6.304<br>**<0.0001**	-0.283 ± 0.096<br>-2.951<br>**0.005**	-0.553 ± 0.096<br>-5.774<br>**<0.0001**	-0.177 ± 0.096<br>-1.848<br>**0.070**	-0.511 ± 0.090<br>-5.653<br>**<0.0001**	-0.096 ± 0.096<br>-1.005<br>**0.310**	-0.130 ± 0.090<br>-1.445<br>**0.154**	0.021 ± 0.090<br>0.235<br>**0.815**	-0.011 ± 0.090<br>-0.124<br>**0.902**					
$J^{0.64}$ HU>CV>HD	0.082 ± 0.090<br>0.903<br>**0.370**	-0.441 ± 0.090<br>-4.879<br>**<0.0001**	-0.154 ± 0.096<br>-1.607<br>**0.113**	-0.425 ± 0.096<br>-4.430<br>**<0.0001**	-0.048 ± 0.096<br>-0.504<br>**0.616**	-0.382 ± 0.090<br>-4.228<br>**0.0001**	0.032 ± 0.096<br>0.339<br>**0.736**	-0.002 ± 0.090<br>-0.020<br>**0.984**	0.150 ± 0.090<br>1.66<br>**0.102**	0.118 ± 0.090<br>1.302<br>**0.198**	0.129 ± 0.090<br>1.426<br>**0.159**				
$J^{0.92}$ HU>CV>HD	0.126 ± 0.096<br>1.316<br>**0.193**	-0.396 ± 0.096<br>-4.135<br>**0.0001**	-0.110 ± 0.101<br>-1.084<br>**0.283**	-0.380 ± 0.101<br>-3.762<br>**<0.001**	-0.004 ± 0.101<br>-0.037<br>**0.971**	-0.337 ± 0.096<br>-3.521<br>**0.001**	0.077 ± 0.101<br>0.762<br>**0.449**	0.043 ± 0.096<br>0.446<br>**0.657**	0.195 ± 0.096<br>2.030<br>**0.047**	0.162 ± 0.096<br>1.692<br>**0.096**	0.173 ± 0.096<br>1.809<br>**0.076**	0.0446 ± 0.096<br>0.465<br>**0.644**			
$J^{0.64}$ HU>HD>CV	0.212 ± 0.090<br>2.351<br>**0.022**	-0.310 ± 0.090<br>-3.431<br>**0.001**	-0.023 ± 0.096<br>-0.243<br>**0.809**	-0.294 ± 0.096<br>-3.065<br>**0.003**	0.083 ± 0.096<br>0.861<br>**0.393**	-0.251 ± 0.090<br>-2.780<br>**0.007**	0.163 ± 0.096<br>1.703<br>**0.094**	0.129 ± 0.090<br>1.428<br>**0.159**	0.281 ± 0.090<br>3.108<br>**0.003**	0.248 ± 0.090<br>2.749<br>**0.008**	0.260 ± 0.090<br>2.873<br>**0.006**	0.131 ± 0.090<br>1.448<br>**0.153**	0.086 ± 0.09583<br>0.900<br>**0.372**		
$J^{0.92}$ HU>HD>CV	0.079 ± 0.090<br>0.879<br>**0.383**	-0.443 ± 0.090<br>-4.903<br>**<0.0001**	-0.156 ± 0.096<br>-1.630<br>**0.108**	-0.427 ± 0.096<br>-4.453<br>**<0.0001**	-0.051 ± 0.096<br>-0.527<br>**0.600**	-0.384 ± 0.090<br>-4.252<br>**0.0001**	0.030 ± 0.096<br>0.316<br>**0.753**	-0.004 ± 0.090<br>-0.044<br>**0.965**	0.148 ± 0.090<br>1.636<br>**0.107**	0.115 ± 0.090<br>1.277<br>**0.207**	0.127 ± 0.090<br>1.401<br>**0.166**	-0.002 ± 0.090<br>-0.024<br>**0.981**	-0.047 ± 0.096<br>-0.488<br>**0.628**	-0.133 ± 0.090<br>-1.472<br>**0.146**	
$J^{0.42}$ HU>HD=CV	0.252 ± 0.096<br>2.631<br>**0.011**	-0.270 ± 0.096<br>-2.820<br>**0.007**	0.017 ± 0.101<br>0.163<br>**0.871**	-0.254 ± 0.101<br>-2.514<br>**0.015**	0.122 ± 0.101<br>1.210<br>**0.231**	-0.211 ± 0.096<br>-2.206<br>**0.031**	0.203 ± 0.101<br>2.010<br>**0.049**	0.169 ± 0.096<br>1.761<br>**0.084**	0.321 ± 0.096<br>3.345<br>**0.001**	0.288 ± 0.096<br>3.007<br>**0.004**	0.299 ± 0.096<br>3.124<br>**0.003**	0.171 ± 0.096<br>1.780<br>**0.080**	0.126 ± 0.101<br>1.247<br>**0.217**	0.040 ± 0.096<br>0.415<br>**0.680**	0.173 ± 0.096<br>1.803<br>**0.077**

$J^{0.42}$ HU>HD=CV

Supplementary model S24: PO_4-P concentration ([PO_4-P], mg L^{-1})

Initial linear regression model:

Lm([PO_4-P] ~ SpD)

No minimal adequate model, intercept only (SpD, $F = 0.57$, d.f. = 15, $p = 0.889$)

Table S1: Summary of data used for statistical analysis. Data in the absence of macrofauna is shown for comparison but was not included in the analyses. SpD = specific arrangements of species dominance, HD = *Hediste diversicolor*, HU = *Hydrobia ulvae*, CV = *Corophium volutator*

SpD	Repli-cate	$^{f\text{-}SPI}L_{mean}$ (cm)	$^{f\text{-}SPI}L_{median}$ (cm)	$^{f\text{-}SPI}L_{max}$ (cm)	SBR (cm)	Δ [Br^-] (mg L^{-1})	[NH_4-N] (mg L^{-1})	[NO_X-N] (mg L^{-1})	[PO_4-P] (mg L^{-1})
$J^{0.42}$ CV>HU=HD	1	2.086	1.718	11.601	1.917	-71.812	3.458	1.048	0.053
$J^{0.42}$ CV>HU=HD	2	3.624	3.405	11.933	1.332	-114.194	0.494	1.220	0.149
$J^{0.42}$ CV>HU=HD	3	2.361	2.366	11.118	0.975	197.692	1.759	0.850	0.013
$J^{0.42}$ CV>HU=HD	4	3.135	3.361	10.999	0.516	-38.074	0.244	0.934	0.060
$J^{0.42}$ CV>HU=HD	5	1.792	1.878	9.531	1.087	32.636	2.009	1.044	0.035
$J^{0.42}$ HD>CV=HU	1	1.919	0.356	10.866	0.701	-7.026	2.895	0.578	0.065
$J^{0.42}$ HD>CV=HU	2	3.073	0.587	11.262	1.024	-13.750	3.044	0.301	0.101
$J^{0.42}$ HD>CV=HU	3	2.061	0.442	10.978	0.885	-32.608	2.424	0.721	0.062
$J^{0.42}$ HD>CV=HU	4	3.207	1.613	11.312	0.570	46.785	3.534	0.489	0.063
$J^{0.42}$ HD>CV=HU	5	1.375	0.412	10.999	0.881	30.107	3.207	0.453	0.071
$J^{0.42}$ HU>CV=HD	1	1.235	0.378	10.951	0.694	52.142	1.942	0.865	0.052
$J^{0.42}$ HU>CV=HD	2	1.288	0.529	10.987	0.609	64.318	1.355	0.740	0.107
$J^{0.42}$ HU>CV=HD	3	1.122	0.578	10.709	0.561	41.718	1.820	0.733	0.074
$J^{0.42}$ HU>CV=HD	4	1.087	0.514	9.949	0.771	-19.256	2.176	0.893	0.040
$J^{0.42}$ HU>CV=HD	5	1.003	0.374	10.759	0.782	-20.893	na	na	na
$J^{0.64}$ CV>HD>HU	1	2.794	2.761	11.337	0.680	56.236	1.832	1.093	0.034
$J^{0.64}$ CV>HD>HU	2	3.316	3.586	11.147	0.961	-4.644	0.681	1.097	0.055
$J^{0.64}$ CV>HD>HU	3	3.246	3.039	10.662	0.876	198.649	0.807	0.926	0.098
$J^{0.64}$ CV>HD>HU	4	3.374	3.482	9.817	0.883	11.597	0.209	0.991	0.051
$J^{0.64}$ CV>HD>HU	5	3.492	3.383	10.908	1.346	-1.422	0.581	1.283	0.044
$J^{0.64}$ CV>HU>HD	1	3.153	3.497	12.000	1.578	-79.117	0.648	1.168	0.024
$J^{0.64}$ CV>HU>HD	2	3.122	3.010	10.576	1.707	-45.670	1.214	1.035	0.052
$J^{0.64}$ CV>HU>HD	3	3.709	3.225	11.721	1.098	-46.438	na	na	na
$J^{0.64}$ CV>HU>HD	4	3.213	3.458	10.079	0.934	-79.149	0.500	0.970	0.069
$J^{0.64}$ CV>HU>HD	5	2.288	2.255	11.265	0.953	-40.154	1.516	1.074	0.057
$J^{0.64}$ HD>CV>HU	1	3.096	2.131	11.640	0.906	-30.025	3.016	0.596	0.052
$J^{0.64}$ HD>CV>HU	2	3.070	1.617	10.701	0.687	-33.481	2.918	0.509	0.065
$J^{0.64}$ HD>CV>HU	3	2.043	0.791	11.146	0.871	402.515	2.880	0.280	0.115
$J^{0.64}$ HD>CV>HU	4	1.980	0.948	11.069	0.851	4.642	1.901	0.329	0.123
$J^{0.64}$ HD>CV>HU	5	2.456	1.582	11.291	0.937	-19.543	2.845	0.722	0.032
$J^{0.64}$ HD>HU>CV	1	2.486	0.886	11.526	0.749	-46.087	2.745	0.454	0.101
$J^{0.64}$ HD>HU>CV	2	1.862	0.551	11.037	0.710	53.278	2.777	0.583	0.069
$J^{0.64}$ HD>HU>CV	3	2.634	0.570	11.044	1.066	450.477	2.695	0.659	0.059
$J^{0.64}$ HD>HU>CV	4	2.860	1.095	10.908	0.607	62.172	3.189	0.723	0.027
$J^{0.64}$ HD>HU>CV	5	0.082	0.000	7.966	0.942	-35.894	na	na	na
$J^{0.64}$ HU>CV>HD	1	2.019	1.558	10.787	0.891	-56.915	2.165	0.523	0.056
$J^{0.64}$ HU>CV>HD	2	1.786	0.720	11.880	1.089	-74.428	1.802	0.805	0.070
$J^{0.64}$ HU>CV>HD	3	2.047	1.372	10.890	0.632	407.152	1.624	0.388	0.111
$J^{0.64}$ HU>CV>HD	4	1.833	0.917	10.755	0.480	3.987	1.923	0.716	0.037

$J^{0.64}$ HU>CV>HD	5	1.894	1.079	11.689	0.767	24.200	1.634	0.754	0.083
$J^{0.64}$ HU>HD>CV	1	1.813	0.708	11.002	0.799	-67.550	2.144	0.758	0.064
$J^{0.64}$ HU>HD>CV	2	2.026	0.880	11.059	0.693	5.874	2.233	0.575	0.096
$J^{0.64}$ HU>HD>CV	3	2.175	0.519	11.163	0.780	77.335	1.740	0.894	0.078
$J^{0.64}$ HU>HD>CV	4	1.578	0.473	11.129	0.564	4.358	2.111	0.652	0.099
$J^{0.64}$ HU>HD>CV	5	1.305	0.556	10.546	0.692	97.362	2.074	0.961	0.035
$J^{0.92}$ CV>HD>HU	1	2.303	2.138	10.728	0.900	483.384	1.979	0.553	0.126
$J^{0.92}$ CV>HD>HU	2	3.490	3.043	11.435	0.658	-2.068	1.312	0.957	0.056
$J^{0.92}$ CV>HD>HU	3	2.741	2.807	11.318	1.360	-0.458	1.465	0.491	0.135
$J^{0.92}$ CV>HD>HU	4	2.559	2.226	11.009	1.113	-38.862	0.671	1.164	0.058
$J^{0.92}$ CV>HD>HU	5	2.853	2.372	11.573	0.869	191.682	na	na	na
$J^{0.92}$ CV>HU>HD	1	2.818	2.622	10.210	0.819	-57.455	1.492	0.630	0.073
$J^{0.92}$ CV>HU>HD	2	2.421	2.328	11.003	1.121	-172.552	1.898	0.617	0.082
$J^{0.92}$ CV>HU>HD	3	2.242	2.071	10.790	1.404	-332.760	2.115	0.761	0.070
$J^{0.92}$ CV>HU>HD	4	2.473	2.377	10.682	0.998	189.481	2.238	0.734	0.043
$J^{0.92}$ CV>HU>HD	5	2.410	2.441	10.704	1.317	37.796	na	na	na
$J^{0.92}$ HD>CV>HU	1	3.436	2.862	11.391	0.922	-49.534	1.949	0.520	0.082
$J^{0.92}$ HD>CV>HU	2	2.551	1.867	10.476	0.674	-121.498	2.228	0.549	0.093
$J^{0.92}$ HD>CV>HU	3	2.513	2.189	11.167	0.449	-29.659	1.595	0.478	0.127
$J^{0.92}$ HD>CV>HU	4	1.677	1.422	11.172	0.771	209.782	2.948	0.416	0.073
$J^{0.92}$ HD>CV>HU	5	2.860	2.199	11.297	0.676	-34.748	2.046	0.635	0.075
$J^{0.92}$ HD>HU>CV	1	2.663	1.831	11.916	0.995	-65.144	1.822	0.723	0.127
$J^{0.92}$ HD>HU>CV	2	2.301	0.945	11.210	0.910	19.606	2.122	0.612	0.096
$J^{0.92}$ HD>HU>CV	3	1.525	0.456	11.414	0.855	-52.193	2.692	0.530	0.080
$J^{0.92}$ HD>HU>CV	4	2.066	0.814	11.307	0.688	35.870	2.721	0.817	0.010
$J^{0.92}$ HD>HU>CV	5	1.909	1.178	11.442	0.780	-72.350	2.845	0.513	0.078
$J^{0.92}$ HU>CV>HD	1	2.453	2.371	10.777	0.684	-12.230	1.386	0.731	0.093
$J^{0.92}$ HU>CV>HD	2	2.104	2.028	11.477	0.666	313.601	1.359	0.547	0.149
$J^{0.92}$ HU>CV>HD	3	2.894	2.927	10.390	1.261	42.820	0.975	0.775	0.050
$J^{0.92}$ HU>CV>HD	4	2.925	3.038	10.068	1.464	-198.486	1.063	0.674	0.046
$J^{0.92}$ HU>CV>HD	5	0.102	0.040	7.677	0.660	2.745	na	na	na
$J^{0.92}$ HU>HD>CV	1	2.073	1.237	10.933	0.840	-41.563	2.433	0.570	0.086
$J^{0.92}$ HU>HD>CV	2	2.023	1.160	11.373	0.602	-27.928	2.034	0.728	0.065
$J^{0.92}$ HU>HD>CV	3	2.378	0.973	11.062	0.398	1.985	2.374	0.636	0.065
$J^{0.92}$ HU>HD>CV	4	2.551	1.790	11.201	0.741	459.424	2.623	0.590	0.030
$J^{0.92}$ HU>HD>CV	5	2.508	1.357	11.241	0.634	162.190	2.213	0.651	0.084
$J^{1.00}$	1	1.884	1.633	10.791	0.573	82.075	2.289	0.593	0.059
$J^{1.00}$	2	2.410	2.038	11.301	0.542	-8.305	2.576	0.438	0.088
$J^{1.00}$	3	2.493	2.206	10.802	1.389	-36.312	2.156	0.612	0.061
$J^{1.00}$	4	3.499	2.545	11.443	1.083	246.772	2.360	0.623	0.059
$J^{1.00}$	5	2.882	2.336	11.083	1.049	45.685	1.103	0.512	0.159
no macrofauna	1	0.110	0.051	3.443	0.623	-23.936	0.285	1.131	0.128
no macrofauna	2	0.070	0.017	0.740	0.510	-14.977	0.238	0.944	0.168
no macrofauna	3	0.088	0.034	5.298	0.547	51.632	0.259	1.342	0.074
no macrofauna	4	0.143	0.000	9.609	0.535	190.181	0.074	1.235	0.062
no macrofauna	5	0.133	0.040	7.714	0.374	-87.699	-0.041	1.038	0.099

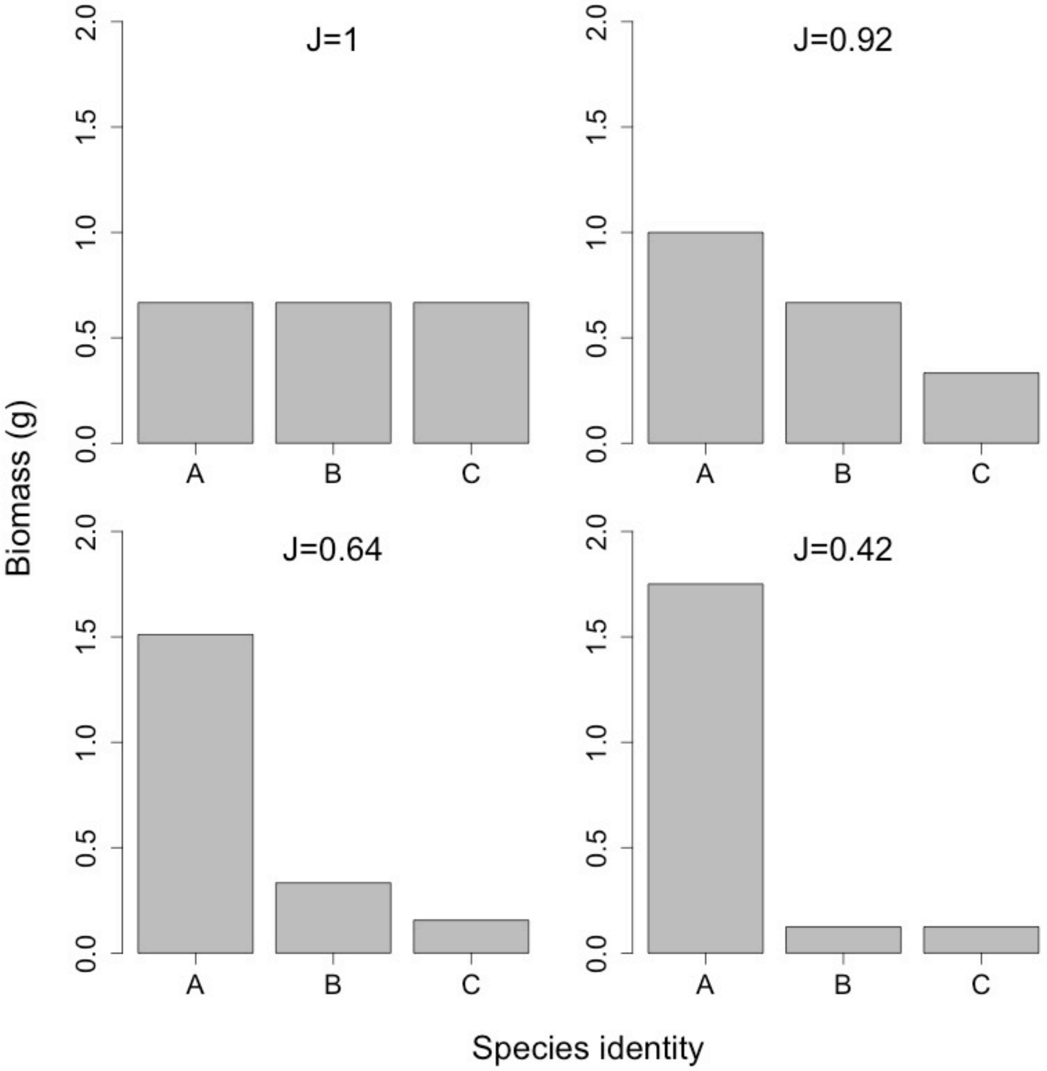

Figure S1: Experimental design: Four arrangements of evenness were selected that span the full spectrum of dominance structures that are possible in natural communities. Each contained three species and each bar represents the biomass of one of the three species: *Hediste diversicolor, Hydrobia ulvae* and *Corophium volutator*. All possible permutations of species dominance rank order where utilised ($J^{1.00}$, 1 permutation; $J^{0.92}$, 6 permutations; $J^{0.66}$, 6 permutations; $J^{0.42}$, 3 permutations).

Table S2: Realised mean (± s.d.) biomass (g) for each permutation of dominance arrangement (n = 5) within each evenness level. HD = *Hediste diversicolor*, HU = *Hydrobia ulvae*, CV = *Corophium volutator.* Realised evenness levels (mean ± s.d.) were: J = 1.00, 0.99 ± 0.0002 (n = 5); J = 0.92, 0.92 ± 0.01 (n = 30); J = 0.64, 0.64 ± 0.01 (n = 30); J = 0.42, 0.42 ± 0.01 (n = 15).

J	HD	± sd	HU	± sd	CV	± sd	total
1.00	0.629	0.029	0.667	0.004	0.653	0.016	1.95
0.92	1.035	0.048	0.666	0.002	0.334	0.005	2.04
0.92	0.994	0.046	0.335	0.003	0.671	0.010	2.00
0.92	0.657	0.040	1.003	0.005	0.336	0.004	2.00
0.92	0.311	0.038	1.003	0.003	0.665	0.003	1.98
0.92	0.320	0.022	0.665	0.002	1.006	0.013	1.99
0.92	0.628	0.020	0.335	0.002	1.019	0.037	1.98
0.64	1.501	0.032	0.334	0.002	0.155	0.003	1.99
0.64	1.525	0.059	0.156	0.002	0.332	0.005	2.01
0.64	0.329	0.011	1.514	0.003	0.157	0.004	2.00
0.64	0.155	0.003	1.512	0.002	0.331	0.008	2.00
0.64	0.317	0.027	0.157	0.001	1.518	0.010	1.99
0.64	0.155	0.005	0.335	0.003	1.515	0.012	2.00
0.42	1.755	0.038	0.125	0.002	0.125	0.004	2.01
0.42	0.118	0.010	1.753	0.002	0.125	0.003	2.00
0.42	0.122	0.005	0.125	0.001	1.749	0.013	2.00

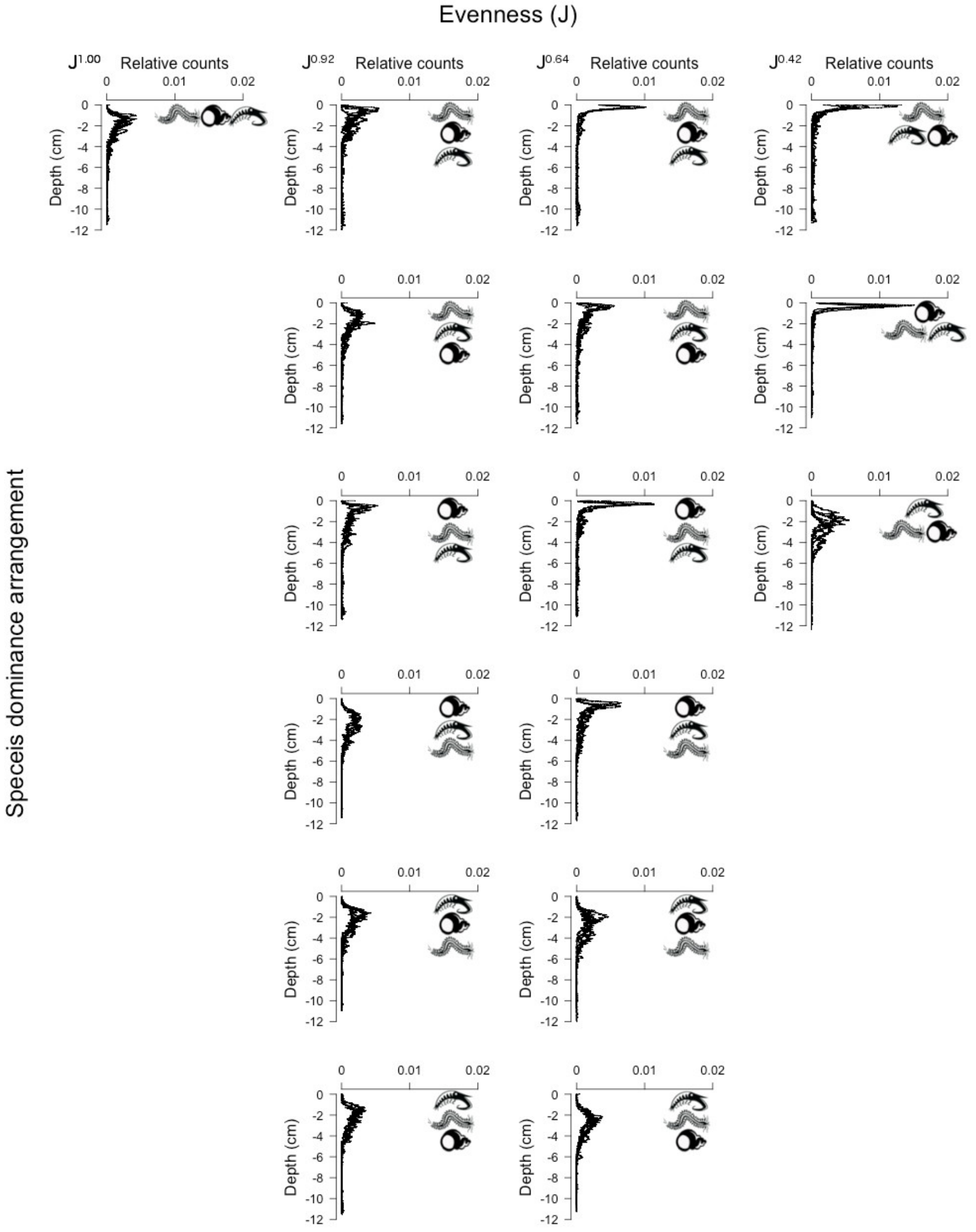

Figure S2: Sediment particle reworking profiles (n = 5) for all arrangements of species dominance across 4 evenness levels ($J^{1.00}$, $J^{0.92}$, $J^{0.64}$, $J^{0.42}$). Relative counts of luminophore tracer particles are standardised (count per depth horizon/total count) to allow comparison between replicates and treatments. The arrangement of species dominance (vertically, from most to least biomass; equal dominance is represented by horizontal positioning and indicates species that share the same amount of biomass) is indicated graphically in the inset of each panel: **=** ***Corophium volutator*****,** = *Hydrobia ulvae***,** **=** ***Hediste diversicolor.***
